# In-silico and in-vitro studies on the efficacy of mangiferin against colorectal cancer

**DOI:** 10.1186/s13065-022-00835-9

**Published:** 2022-06-07

**Authors:** Rohini Samadarsi, Linus Augustin, Chandan Kumar, Debjani Dutta

**Affiliations:** 1 Department of Biotechnology and Biochemical Engineering, Sree Chitra Thirunal College of Engineering, Pappanamcode, Thiruvananthapuram, Kerala India; 2 Department of Biotechnology, National Institute of Technology Durgapur, Mahatma Gandhi Avenue, Durgapur, West Bengal 713209 India

**Keywords:** Autodock Vina, Pharmacophore, PharmaGist, Colon cancer, Cell lines

## Abstract

**Background:**

Mangiferin is a C-glycoside xanthone molecule having a wide range of therapeutic properties. Hence, the present study aims to understand the efficacy of mangiferin against colorectal cancer (CRC) and to elucidate the mechanisms of action of mangiferin on colorectal cancer.

**Method:**

The molecular mechanism of mangiferin against colorectal cancer was studied using Autodock Vina software. Pharmacophore analysis of mangiferin concerning five COX-2 inhibitor drugs was carried out using the PharmaGist server to analyze the possibility of using mangiferin as a COX-2 inhibitor. In vitro analysis of Mangiferin against various cancer cell lines was performed.

**Results:**

The molecular mechanism of action of mangiferin against CRC was assessed by docking with multiple target proteins involved in the progression of CRC. Docking studies showed good binding scores (kcal/mol) ranging from − 10.3 to − 6.7. Mangiferin showed a good affinity towards enzymes like COX-2 and LA4H involved in Arachidonic acid (AA) metabolism with a binding score(kcal/mol) of − 10.1 and − 10.3 respectively. The pharmacophore feature assessment of mangiferin was done for COX-2 inhibitor drugs, which further confirmed that mangiferin poses the same pharmacophore feature as that of COX-2 inhibitor drugs. Furthermore, the binding affinity of mangiferin was compared with five COX-2 inhibitor drugs to prove its efficacy as an inhibitor. Mangiferin also had a cytotoxic effect against colorectal cancer (HT 29), cervical cancer (HeLa), and breast cancer (MCF 7) cell lines. The study could establish that Mangiferin might be a promising candidate for the treatment of colorectal cancer.

**Conclusion:**

In short, these studies exploited the possibility of mangiferin as a lead molecule to develop anticancer/anti-inflammatory drugs for the treatment of CRC.

**Supplementary Information:**

The online version contains supplementary material available at 10.1186/s13065-022-00835-9.

## Introduction

The rise in the incidence of colorectal cancer (CRC) has made it a concerning disease and currently represents approximately 10% of malignant growth-related mortality in western countries. The ‘ascent’ of CRC worldwide is due to the increase in unhealthy dietary propensities, and an increment in hazard factors like smoking, low actual exercise, and obesity [1–10]. CRC is caused by the stepwise interaction of hereditary and epigenetic modifications, leading to the change of ordinary colonic mucosa into intrusive malignant growth [11, 12]. In the CRC, changes might happen either in the oncogenes *K-ras* and APC, or the tumor suppressor gene, p53, causing cell degeneration and uncontrolled cell expansion. Cell multiplication is vital in tumorigenesis and cyclooxygenases (COXs) are significant enzymes in these reactions. COXs catalyzes the conversion of free arachidonic corrosive into prostaglandin H2, which is the antecedent of different prostaglandins and thromboxanes. These compounds play important role in processes like cell multiplication, angiogenesis, resistant capacity, and irritation, which are largely pivotal in the turn of events or movement of neoplasms [8–12].

Advance therapies for primary and metastatic colorectal cancer have emerged, to provide additional options for patients. Although benign colon cancer is often successfully removed by surgery, the high-level illness requires aggressive treatment that has lower efficacy. Around 30 to 75% of patients with colon malignant growth utilize correlative and elective medication (CAM) [1–8]. Many clinical trials suggest that bioactive components from plants in combination with chemotherapy reduced the side effects of chemotherapeutic drugs and improved the survival rates in colon cancer as compared with chemotherapy alone [4–10].

**Fig. 1:**
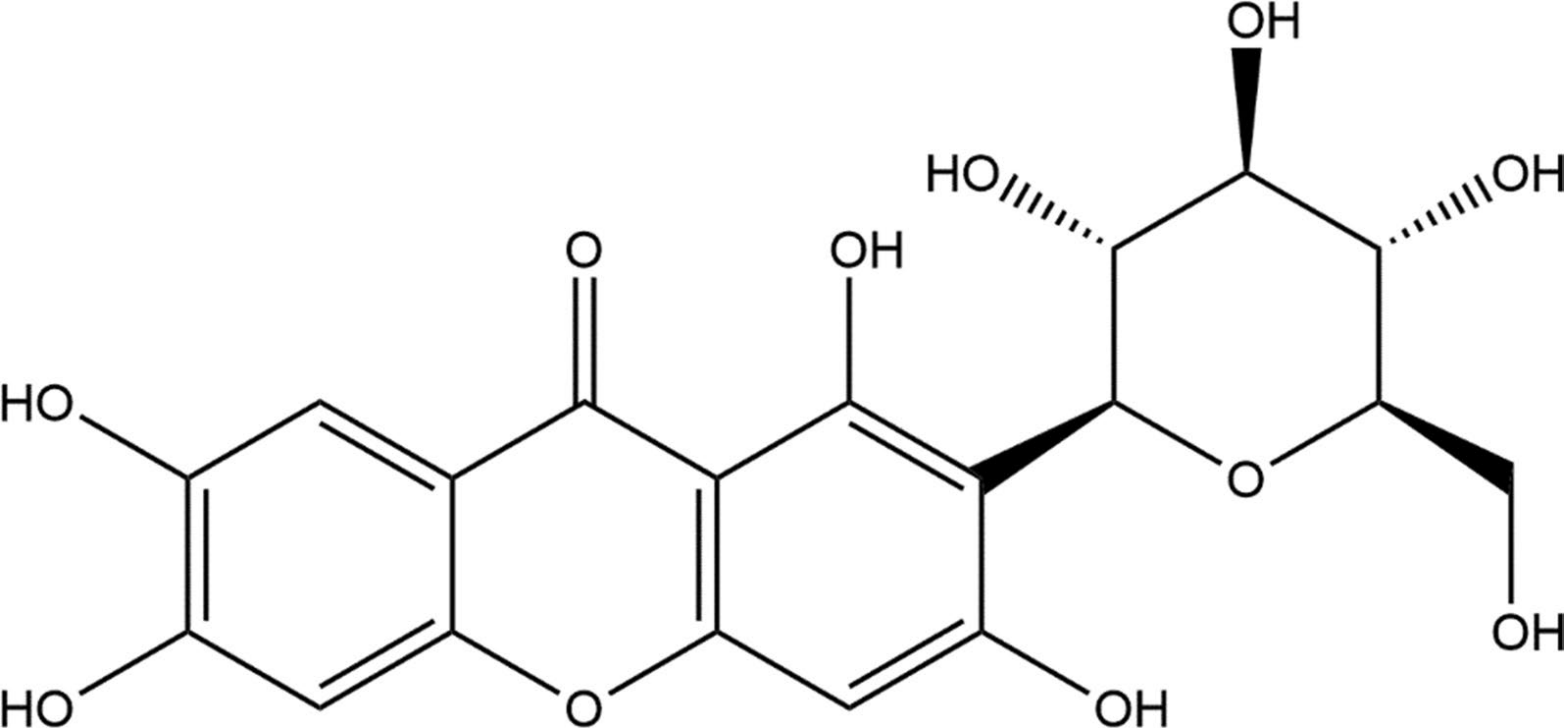
2D structure of mangiferin

Mangiferin (1,3,6,7-tetrahydroxyxanthone-C-2-β-d-glucoside), is a bioactive compound found in a wide variety of plants and is primarily isolated from the stem, bark, and leaves of *Mangifera Indica*. It possesses many health endorsing properties such as antioxidant, antimicrobial, antidiabetic, antiallergic, anticancer, hypocholesterolemic, and immunomodulatory. It suppresses the activation of peroxisome proliferator-activated receptor isoforms by changing the transcription process. Mangiferin protects against different human cancers, including lung, breast, and neuronal cancers, through the suppression of tumor necrosis factor α expression, inducible nitric oxide synthase potential, and proliferation and induction of apoptosis. It also protects against neural and breast cancers by suppressing the expression of matrix metalloproteinase (MMP)-9 and MMP-7 and inhibiting enzymatic activity, metastatic potential, and activation of the β-catenin pathway. It can block lipid peroxidation, to provide a shielding effect against physiological threats [1–3]. The 2D structure of mangiferin is shown in Fig. 1.

Due to the various therapeutic effects of mangiferin, it is used as a drug in Caribbean countries. The Cuban pharmaceutical industry sells mangiferin under the brand name Vimang^®^ [4].

Presently the drugs available in the market for CRC are known to target only a single enzyme/protein [13], for which tumor cells may develop adaptation measures to overcome target protein. Hence, there is an urgent need for novel molecules to combat the severity of colon cancer. One strategy is to identify those naturally occurring bioactive molecules such as mangiferin, that can target multiple enzymes/proteins involved in the progression of CRC, thereby overcoming the limitation of single enzyme/protein-specific inhibitor drugs. Hence in the present study, the binding energy of mangiferin with different types of inflammatory and anti-apoptotic proteins involved in CRC was explored.

Recently computational approaches such as Quantitative structure–activity relationship (QSAR), molecular docking, pharmacophore analysis, and various other approaches have been used for the design and development of novel therapeutic agents that can prevent or inhibit the progression of CRC. Molecular docking is a computational method that is widely used in the drug discovery process, as it provides detailed information about ligand-target complex formation [14]. Docking simulation identifies the best fit conformational pose of ligand that fits well into the active site of the target protein [15]. It also provides detailed information on interacting amino acids between the ligand and target inbound conformation and its binding free energy [14]. Pharmacophore analysis is a method that has been used to identify the structural features such as the geometric arrangement of atoms or functional groups that are responsible for the biological activity of the molecule [16].

This study intended to assess the anticancer property of mangiferin, more precisely against colorectal disease utilizing both in-silico and in-vitro studies. The outcome produced from docking analysis against different liberated colorectal disease proteins showed the versatile mode of action of mangiferin in anticancer treatment. Mangiferin showed a high affinity with the enzymes associated with Arachidonic corrosive (AA) digestion which exhibits its chance as an inhibitor of inflammation (related with metastasis). The Pharmacophore Analysis of mangiferin with COX-2 inhibitor showed a similar mechanism concerning *in-silico* studies. The viability of mangiferin as a COX-2 inhibitor was compared with five economically accessible COX-2 inhibitor medications, and its toxicity investigation was assessed. The cytotoxic impact of Mangiferin was considered in contrast to colorectal disease (HT 29), cervical malignant growth (HeLa), and bosom disease (MCF 7) cell lines. This study investigates the utilization of novel bioactive phytochemicals like mangiferin for the therapy of disease.

## Materials and method

### Softwares

ACD/ChemSketch software, AutoDockTools (ADT), and AutoDock Vina were downloaded from www.scrippps.edu. PyMol, DS Visualizer, and T.E.S.T Software were downloaded from https://pymol.org, http://accelrys.com, and https://www.epa.gov.

### Ligand preparation for docking

The 3D structure of the ligand Mangiferin (CID-5281647) was downloaded from the PubChem Open Chemistry Database (https://pubchem.ncbi.nlm.nih.gov/compound/Mangiferin) and was optimized using AutoDock Tools.

### Preparation of proteins for docking

PDB files of target proteins were obtained from the RCSB protein data bank as given in Table 3. The original structure of proteins was reduced to a unimolecular receptor by using PyMol and modification was brought about by adding polar hydrogens. All the torsional bonds of ligands were set free by the ligand module and gasteiger charges were computed using AutoDock Tools (ADT).

### Determination of binding site

Computed Atlas of Surface Topography of Proteins (CASTp) [17] is a web server used for predicting the active site of the protein. It locates the pockets and voids present in the interior of 3D conformations of proteins and measures the area and volume of the pocket. It also provides detailed information about the number of amino acids and their position involved in the active site [18]. The best binding pockets with high area and volume predicted by CASTp were considered as the potential active sites for docking analysis.

### Docking studies of mangiferin with proteins

Molecular docking simulations were carried out using the AutoDock Vina program to study the interaction between mangiferin and proteins. Using AutoGrid tools, the grid maps were generated in such a way as to cover the active site(s) of the protein predicted by the CASTp. The grid size and grid centers of XYZ points with a grid spacing of 1 Ǻ are listed in the Additional file 2: Table S1. The docking parameter file was set up in the text document. The binding mode was predicted by using the Lamarckian genetic algorithm and the results were analyzed using the binding energies. Default settings were used for all other parameters. The visual analysis of the amino acid of each target protein interacting with mangiferin was done using a DS visualizer. Besides, docking analysis of COX-2 inhibitor drugs concerning the COX-2 enzyme was also evaluated.

### Pharmacophore analysis

Pharmacophoric features were generated with the aid of a web-based freely available pharmacophore identification server Pharmagist [19]. The MOL2 file of five COX-2 inhibitor drugs whereas uploaded to the server and all the settings were kept as default. The server constructs a pharmacophore using six different features—H-bond acceptor, H-bond donors, aromatic centers, hydrophobic centers, negative charge, and positive charge.

### Prediction of toxicity profiling by computational analysis of mangiferin

The toxicity profiling of Mangiferin was determined by using The Toxicity Estimation Software Tool (TEST) allows the user to easily estimate the toxicity of chemicals using Quantitative Structure–Activity Relationships (QSARs) methodologies. QSARs are mathematical models used to predict measures of toxicity from the physical characteristics of the structure of chemicals (known as molecular descriptors) [20–26].

### Cells and culture conditions

Mangiferin standard was purchased from Sigma and the required concentrations were prepared fresh in RPMI1640 containing 10% fetal calf serum on the day of use [5–8].

### Cell lines

Three human cancer cell lines, HeLa (cervical cancer), HT29 (colon cancer), and MCF7 (breast cancer), were maintained in 10 cm culture dishes (Nunc) at 37 °C in a humidified incubator containing 5% CO_2_ in growth medium (RPMI1640 supplemented with 10% fetal calf serum) [5–8].

### In vitro cytotoxicity assay and dose–response curves

Dose–response curves were performed by using the MTT assay (Sigma, USA) and the IC_50_ values were calculated using the Origin 7.5 software (Origin Lab Corp., MA, USA). Cells (200 µl per well) were seeded in flat-bottom 96 well culture plates (Nunc) at 30 000 cells/ml and incubated overnight at 37 °C in a humidified incubator containing 5% CO_2_. Cells were allowed to attach and recover for 24 h before mangiferin was added to the wells at concentrations of 5, 10, 15, and 30 µg/ml A stock solution of MTT was prepared in PBSA (5 mg/ ml) and further diluted to 0.5 mg/ ml with growth medium. The medium from each well was replaced with 200 µL MTT solution before the plates were incubated for another 3 h. MTT solution was replaced by 200 µL DMSO and absorbance was taken at 540 nm on a Lab systems Multiskan MS Plate Reader. Viable cell percentage was calculated using the following equation (Eq. (1)) [27].

1 $$Cell Viability\left(\%\right)=\frac{\mathrm{OD of treated cells}}{\mathrm{OD of untreated cells}}\mathrm{X}100$$

The IC_50_ (concentration causing 50% inhibition of cell growth) values were obtained from the results of triplicate determinations of at least three independent experiments.

### Statistical analysis

In the present work, samples were analyzed in triplicate batches, and in means ± standard deviations of three determinations, the data are presented. Statistical differences between the two groups were evaluated using the Student’s t-test and multiple comparisons of means were calculated by LSD (least significant difference) test. A probability value of < 0.05 was considered significant. All statistical analysis was performed using the Origin 7.5 software (Origin Lab Corp., MA, USA) [27].

## Results and discussions

### Molecular docking

In the present study, the binding energy of mangiferin with different types of inflammatory and anti-apoptotic proteins involved in CRC was explored and the In-silico analysis to understand the mechanism of interaction of mangiferin with these proteins was carried out. The docking scores of mangiferin with these selected proteins are summarized in Table 1. The best binding pockets with high area and volume predicted by CASTp were considered as active sites for each protein (Additional file 2: Table S2).

**Table 1:** Docking results for the Mangiferin-protein interaction

Sl. No.	Protein receptors	PDB ID	Binding energy (kcal/mol)
1	APC	3NMZ	−8
2	BUBR1	3SI5	−6.7
3	CDK8	3RGF	−8.1
4	CK2α	3WAR	−8.3
5	FABP6	5L8I	−8.8
6	K-Ras	4OBE	−8.6
7	Spindle assembly checkpoint protein human MAD2	1DUJ	−7.6
8	Bcl-xL	1MAZ	−7.4
9	Bcl-2	2XA0	−7.6
10	COX-2	3NT1	−10.1
11	CYTOCHROME P450	4NZ2	−8.7
12	PROTEIN KINASE B	1UNR	−7.7
13	TNFα	4TWT	−7.6
14	LEUKOTRIENE A4 HYDROLASE	1HS6	−10.3
15	NF-қB	1VKX	−8.2

**Fig. 2:**
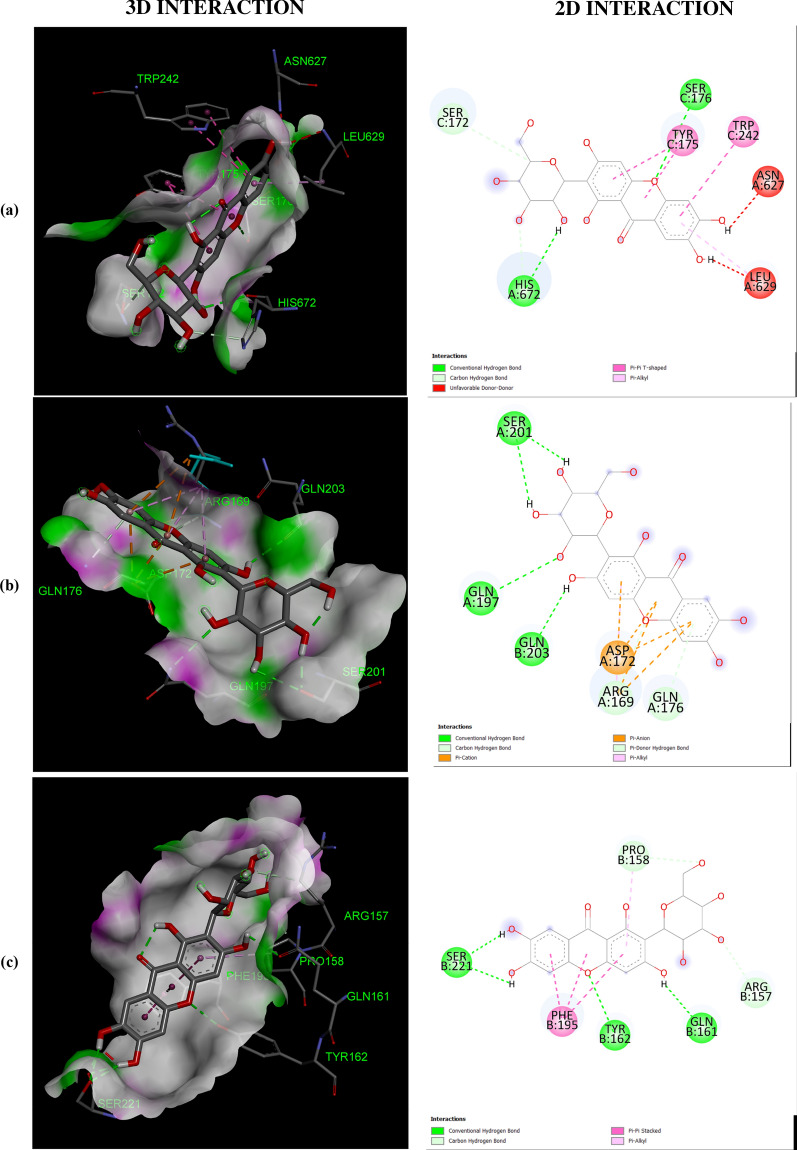

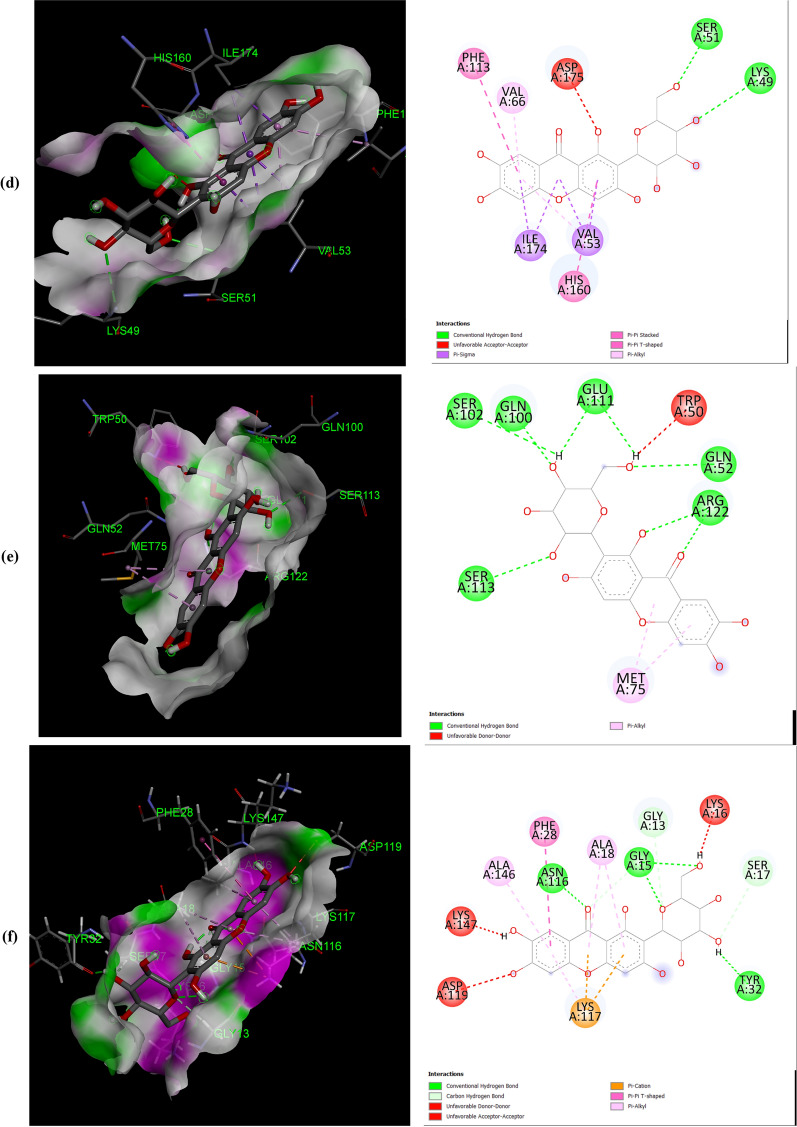

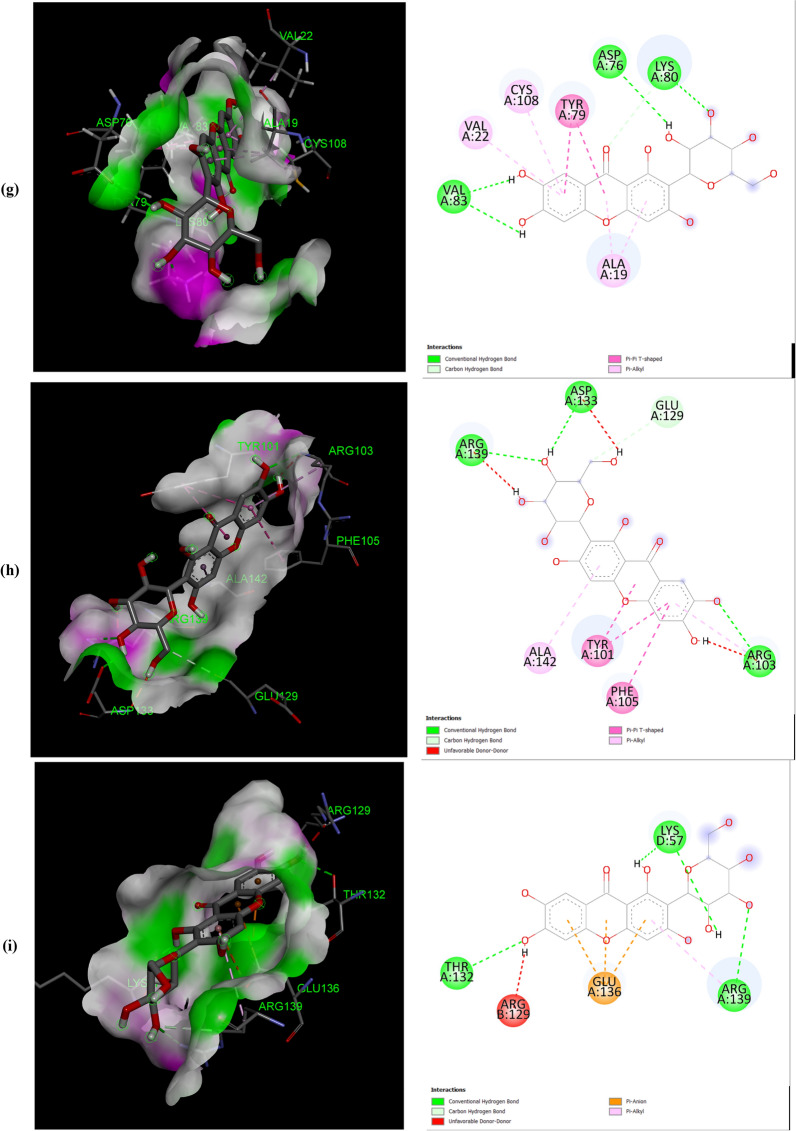

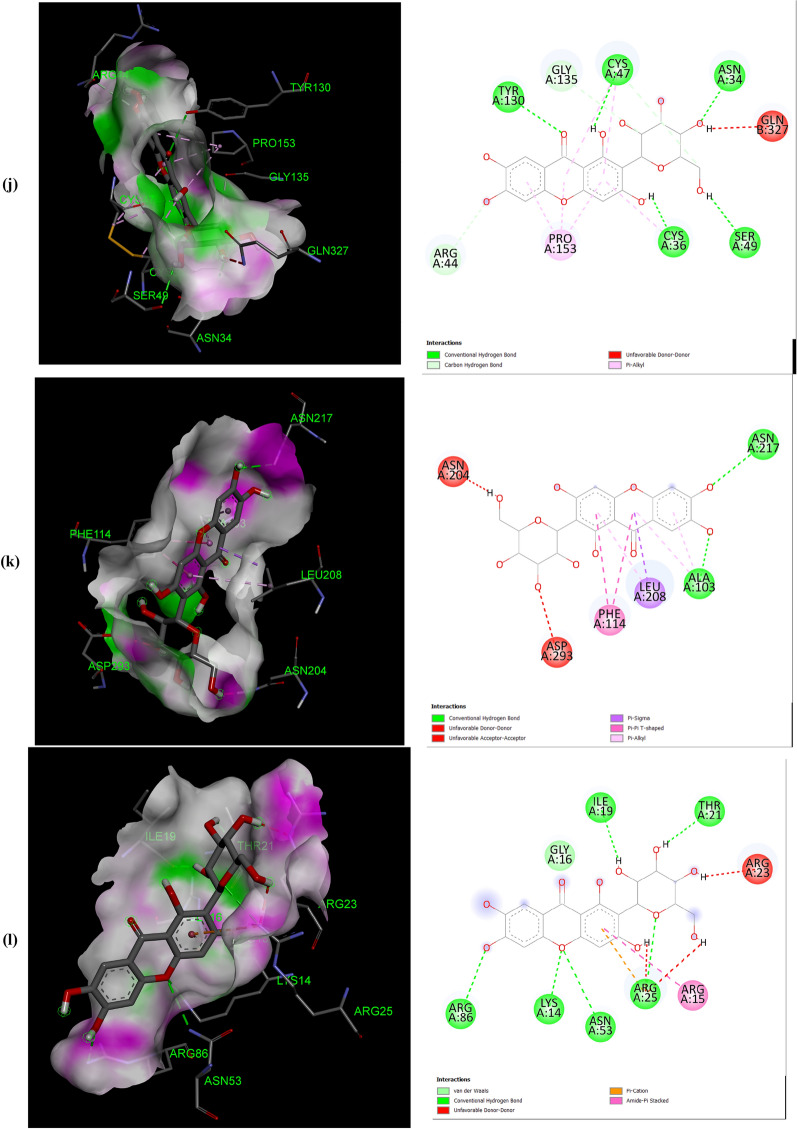

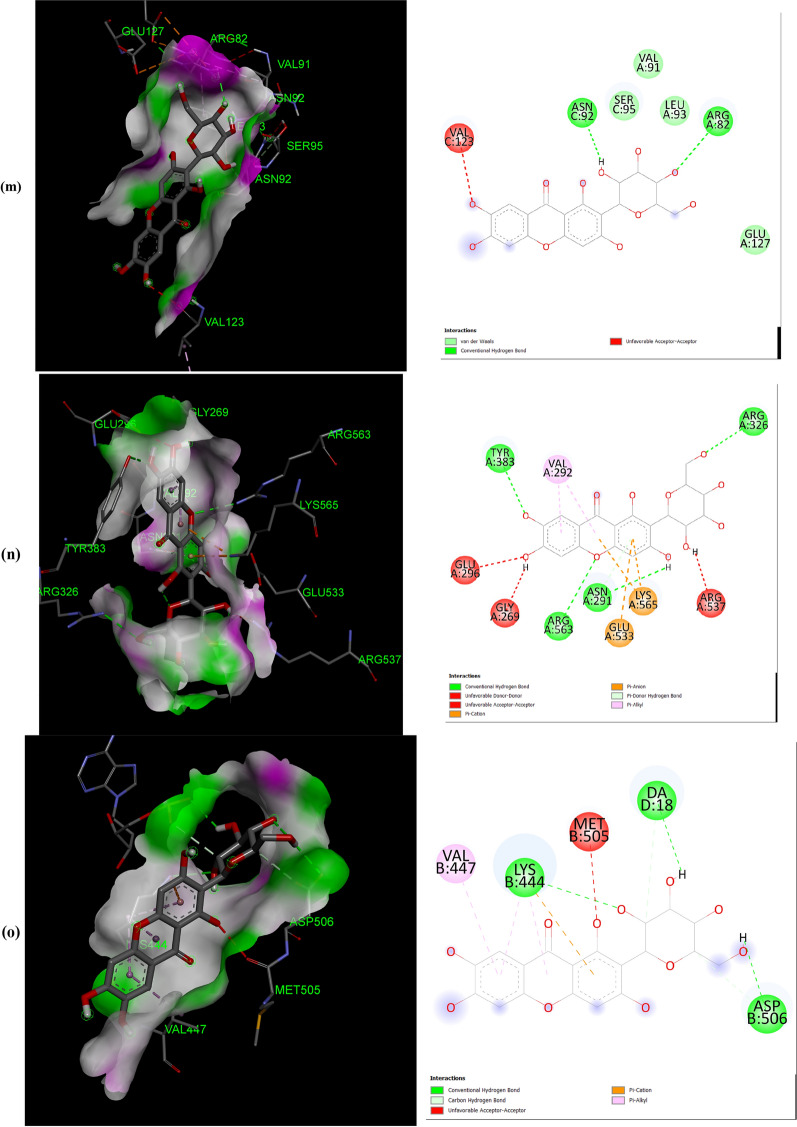
Interaction of Mangiferin with: **a** APC protein; **b** BUBR1protein; **c** CDK8 protein; **d** CK2α protein; **e** FABP6 protein; **f** K-Ras protein; **g** SPINDLE ASSEMBLY CHECKPOINT PROTEIN HUMAN MAD2 protein; **h** Bcl-xL protein; **i** Bcl-2 protein; **j** COX-2 protein; **k** CYTOCHROME P450 protein; **l** PROTEIN KINASE B protein; **m** TNFα protein; **n** LEUKOTRIENE A4 HYDROLASE protein; **o** NF-қB protein

The negative binding energy of the mangiferin-protein complex showed the ability of mangiferin to form a stable interaction with selected proteins. Mangiferin forms a stable complex with leukotriene A4 hydrolase (LA4H) and COX-2 with a binding score (kcal/mol) of − 10.3 & − 10.1. Mangiferin creates hydrogen bonds and other non-interactive bonds in the active pockets of the above-listed proteins (Fig. 2). All the selected proteins showed a good binding score (kcal/mol) ranging from − 10.3 to − 6.7 confirming binding with mangiferin.

The help of computational biology led to the pathway of new improved and novel therapeutic discovery [28]. The water repellent section of amino acids Leu83 and Asp146 play a crucial role in CDK8 regulation [29], which acts as a pharmaceutical target for cancer treatment. The hydrogen bond structure of cyclin B/CDK2 and dockings core indicated that mangiferin could effectively suppress the activity of COX-2 protein. Amino acids His95 and Val96 play an important role in activating COX-2 [17]. The post-scoring approach was evaluated by using the docking complexes. The free energy computation process obtained by MM/GBSA led to the consolidation of molecular logistics intensity and constant resolver modes. In terms of hydrogen and non-hydrogen bond interactions with protein–ligand complex, such as van der Waals, electrostatic energies, polar and non-polar destruction free forces with the supplement form of entropy. Thus, these proteins were concluded as receptors to explore the anti-cancerous activity of selected bioactive components [18]. From the current work, it was observed that the binding affinity of COX-2 was higher with mangiferin when compared to the other proteins APC BUBR1, CDK8, CK2α FABP6, K-Ras, SPINDLE ASSEMBLY CHECKPOINT PROTEIN HUMAN MAD2, Bcl-xL, Bcl-2, CYTOCHROME P450, TNFα, LEUKOTRIENE A4 HYDROLASE, and NF-қB (Table 1). Hence, it could be inferred that mangiferin would have better inhibition activity with COX-2 protein and mangiferin could be taken as a potent inhibitor of COX-2 protein.

Mangiferin was also docked with COX-2 protein deleting the bound inhibitor Rofecoxib (Vioxx), which is a well-known inhibitor of COX-2 protein, sharing the same binding site. It was observed that mangiferin, still had a very good binding affinity towards the COX-2 protein (Data shown in the Additional file 1). In our experience, this is the first report to explore the anti-cancer activity of mangiferin against Colon cancer through molecular docking analysis.

The number and position of amino acids (in the active pocket of proteins) interacting with mangiferin are summarized in Table 2. Docking studies showed that mangiferin binds more efficiently with amino acids having nonpolar R-group, polar uncharged R-group, and positively charged R-group. It also showed maximum binding with aromatic amino acids but least binding with negatively charged R-group amino acids. More frequent interaction of mangiferin was shown with lysine, arginine, and asparagine, and least interaction with methionine and isoleucine (Table 3).

**Table 2:** Interacted amino acid residues of proteins with Mangiferin

Protein receptors	Interacting amino acid residue(S)
APC	ASN627 (2), HIS672 (2), THR628, SER172, LEU629 (2), GLU633, TYR175 (2), TRP242 (2)
BUBR1	SER201, THR204, ASP172 (3), GLN176, ARG169 (4)
CDK8	TRP6 (2), ARG157 (3), TYR162, GLN161, PRO158 (2), PHE195 (3)
CK2α	LEU45 (4), ASN118 (2), SER51, HIS160 (2), VAL53 (3)
FABP6	GLN52 (2), TYR98, SER113, ARG122 (2), GLU111, MET75 (2)
K-Ras	GLY15 (3), LYS16, SER17, TYR32 (2), ASN116, PRO34, LYS147, LYS117 (4), PHE28, ALA18 (2)
Spindle assembly checkpoint protein human MAD2	ALA112, ASP76, CYS81, VAL83, LYS80, SER82, ALA19 (2), TYR79 (2), VAL22, CYS108
BCL-xL	PHE105 (2), ARG139, GLU129, ARG103 (2), ASP133, TYR101 (2), ALA142
Bcl-2	LEU59 (2), SER60 (2), GLU136, GLU61, VAL133 (2), LYS58 (3)
COX-2	ASN34 (2), PRO154, CYS47 (3), CYS36 (2), ARG44 (2), GLY135, PRO153 (3)
CYTOCHROME P450	ASN217 (2), VAL292, ASP293, LEU208 (2), PHE114 (2), ALA103 (2)
PROTEIN KINASE B	LYS14 (2), THR21 (2), ASN53, ILE19, GLU17
TNFα	ASN92, GLN125, LEU93
LEUKOTRIENE A4 HYDROLASE	ARG563 (2), LYS565 (2), GLU501, ASN341 (2), GLU296, VAL292
NF-қB	HIS405, SER471, ASN403, LEU467 (2), SER471, ILE493, ALA497 (3)

**Table 3:** The number of times amino acid interacts with Mangiferin

Proteins receptors	GL Y	ALA	P RO	VAL	LE U	I LE	ME T	P HE	T YR	T RP	S ER	T HR	C Y S	A SN	G LN	L Y S	A R G	HI S	A SP	G LU
APC					+				+ +	+ +	+	+		+ +				+ +		+
BUBR1											+	+			+		+ + + +		+ + +	
CDK8			+ +	+ + +					+	+ +					+		+ + +			
CK2α				+ + +	+ + + +						+			+ +				+ +		
FABP6							+ +		+		+				+ +		+ +			+
K-Ras	+ + +	+ +	+					+	+		+			+		+ + + + + +				
SPINDLE ASSEMBLY CHECKPOINT PROTEIN HUMAN MAD2		+ + +		+ +					+ +		+		+ +			+			+	
Bcl-xL		+						+ +	+ +								+ + +		+	+
Bcl-2				+ +	+ +						+ +					+ + +				+ +
COX-2	+		+ + + +										+ + + + +	+ +			+ +			
CYTOCHROME P450		+ +		+	+ +			+ +						+ +					+	
PROTEIN KINASE B						+						+ +		+		+ +				+
TNFα					+									+	+					
LEUKOTRIENE A4 HYDROLASE				+										+ +		+ +	+ +			+ +
NF-қB		+ + +			+ +	+					+ +			+				+		
Total frequency	4	11	7	12	12	2	2	5	9	4	10	4	7	14	5	14	16	5	6	8

+ Number of times Mangiferin interacts with respective amino acid

All the proteins selected for docking analysis are associated with molecular pathways having a specific role in the progression of CRC. Docking results showed that mangiferin can strongly inhibit the inflammatory pathway (5-LOX and COX-2 pathway) associated with Arachidonic acid (AA) metabolism. Leukotriene A4 hydrolase (LTA4H) is a bifunctional zinc enzyme involved in the 5-LOX pathway which catalyzes the biosynthesis of leukotriene B4, a classical chemo-attractant and immune-modulating lipid mediator. This enzyme is overexpressed in colon cancer and is therefore regarded as a relevant target for cancer therapy [30]. A study conducted by Jeong et al. showed that the inhibition of this protein suppressed the tumor progression of CRC [31]. Hence, in the present study mangiferin was docked with LTA4H protein with a binding value of − 10.3 kcal/mol, suggesting the possibility of using mangiferin as a potential compound in the treatment of CRC. Similarly, mangiferin was docked with COX-2, an inducible enzyme that regulates prostaglandin synthesis, and was found to be overexpressed at sites of inflammation and colon cancer. This enzyme plays a significant role in the regulation of apoptosis, angiogenesis, and tumor cell invasiveness. Selective inhibitors of COX-2 are employed to regress colorectal polyps in CRC patients [32]. So there is a need for developing novel agents that could surpass the activity of the COX-2 enzyme. Docking results showed that mangiferin can form a stable complex with COX-2 enzyme with a favorable binding score of − 10.1 kcal/mol predicting the possibility of using mangiferin as a COX-2 inhibitor.

Aberrant activation of numerous molecular pathways (such as Wnt/β-catenin, Akt, NF-κB, and TNF Signaling pathways) are seen in CRC which causes its progression [33–37]. Therefore docking analysis of mangiferin was carried out with key proteins (APC, Cyclin-Dependent Kinase 8 (CDK8) K-Ras, Protein Kinase B (Akt kinase), Nuclear factor-κB and Tumor Necrosis Factor-α (TNF-α) associated with these pathways, and was found that mangiferin formed a stable complex with binding energy ranging from − 8 to − 7.6 kcal/mol indicating the possibility of mangiferin acting as an inhibitor. Anti-apoptotic proteins such as B-cell lymphoma 2(Bcl-2) and Bcl-xL protein are found to be overexpressed in CRC [38] and were docked with mangiferin. The docking results showed that mangiferin can block the activity of these anti-apoptotic proteins with a binding score of − 7.6 and − 7.4 kcal/mol, indicating the possibility that mangiferin can increase the rate of cancer cells undergoing apoptosis.

BUBR1and MAD2 proteins are key elements of the mitotic checkpoint complex which monitors the cell cycle progression. Mutations in the gene coding for these proteins are seen in CRC causing chromosome instability [39, 40]. These proteins were docked with mangiferin and the binding score was found to be − 6.7 kcal/mol and − 7.6 kcal/mol. This shows the possibility of using mangiferin in the treatment of CRC by targeting proteins involved in the Spindle Assembly Checkpoint (SAC). Similarly, CK2α protein kinase, an enzyme that participates in the regulation of the cell cycle was found to be overexpressed in CRC [41]. Mangiferin was docked with this protein which showed a favorable binding score of − 8.3 kcal/mol indicating that mangiferin can inhibit the activity of CK2α protein kinase.

Mangiferin was also docked with FABP6 and Cytochrome P450. FABP6 is a bile acid-binding protein that transports bile acid to ileal epithelial cells [42] and was overexpressed in CRC. Docked result of mangiferin with FABP6 showed a favorable binding score of − 8.8 kcal/mol indicating that mangiferin can act as an inhibitor in the transport of bile acid to colon mucosa, thereby restricting the progression of CRC. Cytochrome P450 plays a role in the oxidative metabolism of a wide range of xenobiotics and biologically active endogenous compounds [43]. Recently it was found that P450s play a major role in tumor development via oxidative metabolism of carcinogens [44]. So the development of P450 inhibitors can suppress tumor progression [45]. Docked result of mangiferin concerning Cytochrome P450 showed a favorable binding score of − 8.7 kcal/mol. In-silico analysis of mangiferin with these target proteins suggests that mangiferin has a broad spectrum of anticancer mechanisms which makes it a potential candidate for the development of the novel drug in the treatment of cancer, especially CRC. In our experience, this is the first report to explore the anti-cancer activity of mangiferin against Colon cancer through molecular docking analysis.

### Pharmacophore analysis

COX-2 is a crucial protein/enzyme in prostanoid synthesis and development, which acts as the mediator of inflammation and the immune system and can potentiate the infiltration rage and cellular transmission through the membrane of vessels´ walls. Various scientific reports have proved the involvement of COX-2 in the development of inflammation, cancer, and neurodegenerative diseases. Numerous studies prove the correlation between inflammation and cancer. The intake of nonsteroidal anti-inflammatory drugs (NSAIDs) which inhibit COX-2 activity reduces the number of inflammatory mediators, further resulting in a lower incidence of cancer. Hence in the present study pharmacophore was designed as per NSAID drugs. Food and Drug Administration (FDA) has approved Cox-2 inhibitors for the treatment of colon cancer [46]. Pharmacophore features analysis of mangiferin concerning five COX-2 inhibitor drugs—Vioxx, Bextra, Nimesulide, indomethacin, and Celebrex [40, 46] confirmed the potential of mangiferin as a COX-2 inhibitor, which aided in support with the docking analysis. The pharmacophore model was generated by the pairwise alignment by considering six different features such as H-bond acceptors, H-bond donors, aromatic centers, hydrophobic centers, negative charge, and positive charge (Table 4, 5).

**Table 4:** Data set displaying the pharmacophore feature of COX 2 inhibitor drugs along with Mangiferin

Molecule	Atoms	Features	Spatial features	Aromatic	Hydrophobic	Donors	Acceptors	Negative	Positive
Bextra	36	15	14	2	7	1	5	0	0
Celebrex	40	17	16	1	10	1	4	0	1
Nimesulide	33	9	9	2	1	1	5	0	0
Indomethacin	40	12	12	2	4	0	4	1	1
Vioxx	36	8	8	2	2	0	4	0	0
Mangiferin	48	22	14	3	0	8	11	0	0

**Table 5:** Best fit alignment score obtained using PharmaGist

Score	Features	Spatial features	Aromatic	Hydrophobic	Donors	Acceptors	Negative	Positive	Molecules
The best fit alignment score of COX 2 inhibitor drugs									
11.906	3	3	0	0	0	3	0	0	Bextra, celebrex, nimesulide, indomethacin, vioxx
Best fit alignment score of COX 2 inhibitor drugs + Mangiferin									
12.728	3	3	0	0	0	3	0	0	Bextra, celebrex, nimesulide, indomethacin, vioxx, mangiferin

**Fig. 3:**
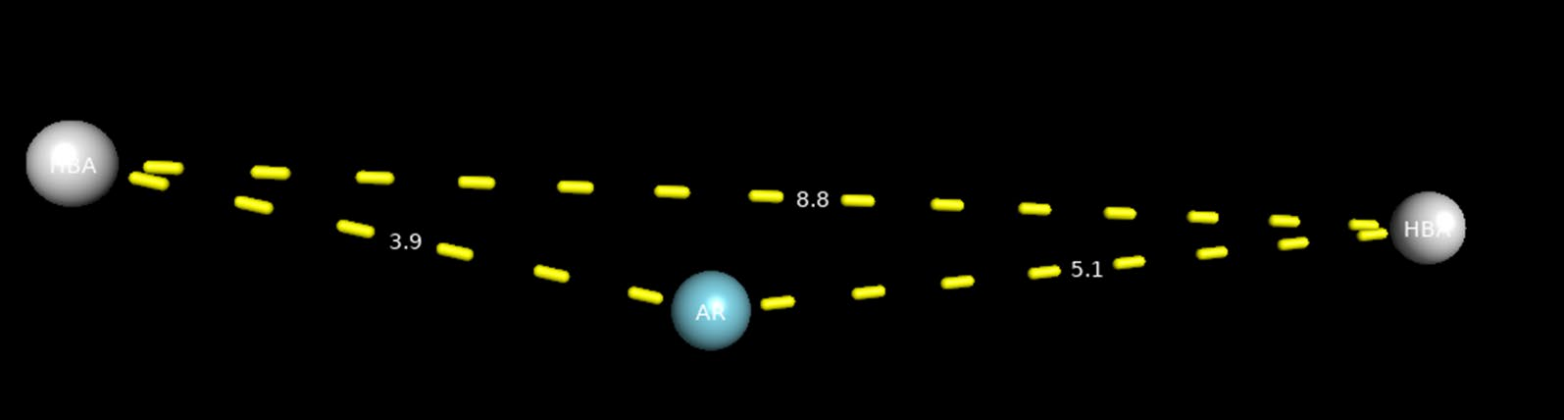
Common pharmacophore model generated by the alignment of 5 COX-2 inhibitors along with/without mangiferin

**Fig. 4:**
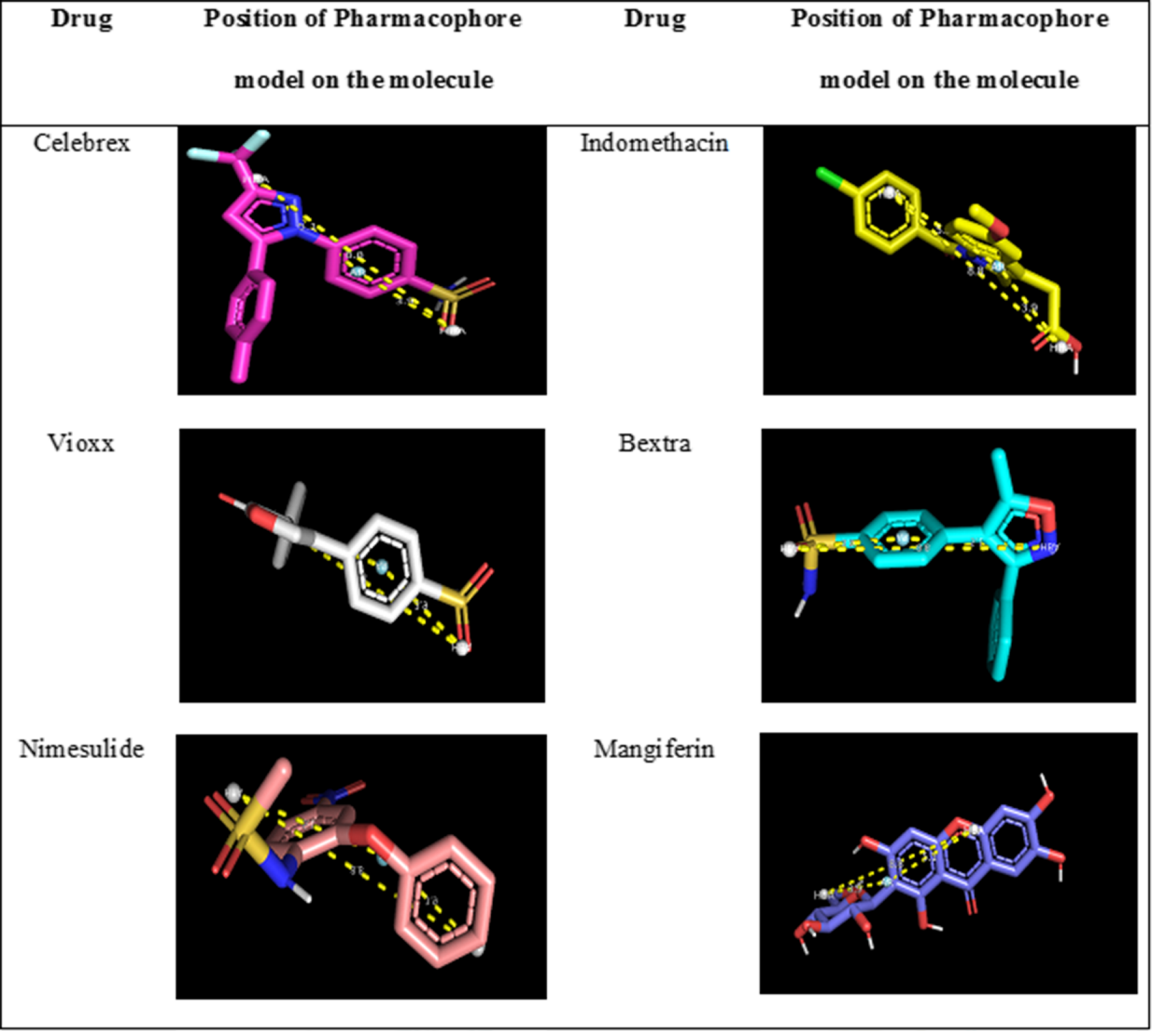
Position of the pharmacophore model on the molecule

One of the generated models for the five COX-2 inhibitor drugs was found to be identical with the model generated when mangiferin was incorporated in the data set (Fig. 3). This suggests that mangiferin can act as a COX-2 inhibitor as it possesses the same pharmacophore features—two hydrogen bond acceptors (HBA) and one aromatic moiety (AR), that are seen common in all the five COX-2 inhibitor drugs. (Fig. 4).

Mangiferin was docked into the crystal structures of COX-1/2 (PDB ID: 4M11, 4O1Z) for validation of the docking process. Since mangiferin binds to COX-1/2 using two water molecules situated on each side of the ligand [27], waters 84 and 161 in COX-2 were retained to obtain an unequivocal pose concerning co-crystal configuration. The Induced Fit Docking (IFD) protocol reproduced well the interaction confirmation of mangiferin with root mean squared deviation (RMSD) values of 1.407 Å (COX-2) (Fig. 4 and Tables 4, 5). Excepting a direct hydrogen bond interaction, the mangiferin does not interact directly with binding site amino acid residues (Table 3). Particularly, mangiferin makes two hydrogen bonding networks with two highly coordinated water molecules to Tyr385/Ser530 (water 25 (COX-2) and Arg120/Tyr355 (water 84 (COX-2)) (Data given in Additional files 1, 2).

Further, the binding energy score of these five COX-2 inhibitor drugs has been assessed and compared with the binding score of mangiferin. (Table 6). A limited number of studies have shown the impact of NSAIDs in the prevention of tumors in humans. NSAIDs block endogenous prostaglandin synthesis through inhibition of COX enzymatic activity. Overexpression of COX-2 is observed in various cancers. There are two COX enzymes, one predominating at sites of inflammation (COX-2) and one constitutively expressed in the gastrointestinal tract (COX-1), which has led to the important therapeutic development of COX-2 inhibitors. COX-2 is phylogenetically more primitive than COX-1 and, while very similar, has critical differences, particularly the existence of a small pocket halfway down the active enzyme site. Several drugs achieve selectivity by binding to this pocket, including presumptively Bextra and Celebrex. However, there are other drugs, such as meloxicam, which inhibit COX-2 by binding to different pockets [12].

**Table 6:** Docking analysis of mangiferin and COX-2 inhibitor drugs

Drug Name	Binding Energy (Kcal/mol)
Mangiferin	− 10.1
Bextra	− 8.7
Celebrex	− 9.0
Vioxx	− 8.8
Indomethacin	− 7.8
Nimesulide	− 8.2

In the present study, mangiferin could successfully bind to the catalytic core of the COX-2 enzyme. Catalytic core of Cox-2 Enzyme consists of the following amino acids namely, His75, His90, Val116, Leu117, Arg120, Gln178, Gln192, Phe205, Phe209, Val335, Leu338, Ser339, Tyr341, Val344, Ile345, Tyr348, Val349, Leu352, Ser353, Tyr355, Leu359, Tyr371, Trp373, Phe381, Leu384, Tyr385, Trp387, Arg499, Ala502, Phe504, Val509, Gly512, Ala513, Ser516, Phe518, Met522, Val523, Gly526, Ala527, Ser530, Leu531, Gly533, Leu534.

In POCKET 01 (His90, His95), Mangiferin was found to bind with either His90 or His95 or both via H-bond. In POCKET 02 (Val116, Leu117, Arg120), Mangiferin was also found to bind with Arg120 via H-bond and in some cases, mangiferin was also found to bind with Val116. POCKET 03 (Phe205, Phe209) Mangeferin was unable to bind in this pocket with negative ∆G. POCKET 04 (Val344, Ile345, Tyr348, Val349, Leu352, Ser353, Tyr355, Leu359), Most of the orientations of mangiferin were found to bind with Tyr355, Arg120 via H-bond and in some cases mangiferin found to bind with Val349, Leu352, Leu359. POCKET 05 (Phe381, Leu384, Tyr385, Trp387, Phe518, Met522, Val523, Gly526, Ala527, Ser530, Leu531, Gly533, Leu534), Most of the orientations of mangiferin were found to bind with Met522, Tyr385, Ser530 via H-bond and in some cases mangiferin was also found to bind with Tyr355, Arg120, Ala527, Val349. Hence it could be inferred that mangiferin could block the activity of the COX-2 enzyme. (Data are shown in Additional files 1, 2).

It is already well established the direct correlation between that COX-2 and cancer development. Hence the treatment with COX-2 inhibitors might relieve their symptoms and limit their adverse effects of colon cancer. Thus COX-2 inhibitors would be a safe and effective compound that could present different properties such as anti-inflammatory, antiplatelet, and anticancerous effects [46].

The results showed that mangiferin has a higher binding affinity than the five COX-2 inhibitor drugs, which makes a potential lead molecule a COX-2 inhibitor (Table 6), which could be useful in the treatment of inflammation associated with colon cancer.

### Toxicity analysis of Mangiferin

The toxicity profiling is an essential step in the drug development process, and therefore by using T.E.S.T software, toxicity analysis of mangiferin was carried out. The software uses the structural information to predict the toxicological properties of the compound and compares it with “similar” chemicals for developing local QSAR models [20]. Table 7 contains the oral rat LD50 (mg/Kg) and Bioaccumulation factor predicted for mangiferin using T.E.S.T software.

**Table 7:** Predicted toxicological analysis of Mangiferin

Parameter	Predicted value (by consensus method)
Oral rat LD_50_ (mg/Kg)	5616.04
Bioaccumulation factor	0.40

The consensus method was employed to calculate the toxicity properties of mangiferin. This method estimates the properties based on results obtained from several approaches- the Hierarchical method, FDA method, Single-model method, Group contribution method, and the Nearest neighbor method [20].

The LD50 value of mangiferin was calculated and predicted to be non-toxic, as the quantity required to kill 50% of the dose group of rats was found to be around 5616.04 mg/kg which comes under the category of “Practically Non-toxic Group”[51]c (Additional files 2: Table S5). Bioaccumulation factor (BAF) gives an idea about the possibility of accumulation toxicity of chemicals within the body and mangiferin was predicted to be not bioaccumulative as the value was found to be 0.4.

### Cytotoxicity

**Fig. 5:**
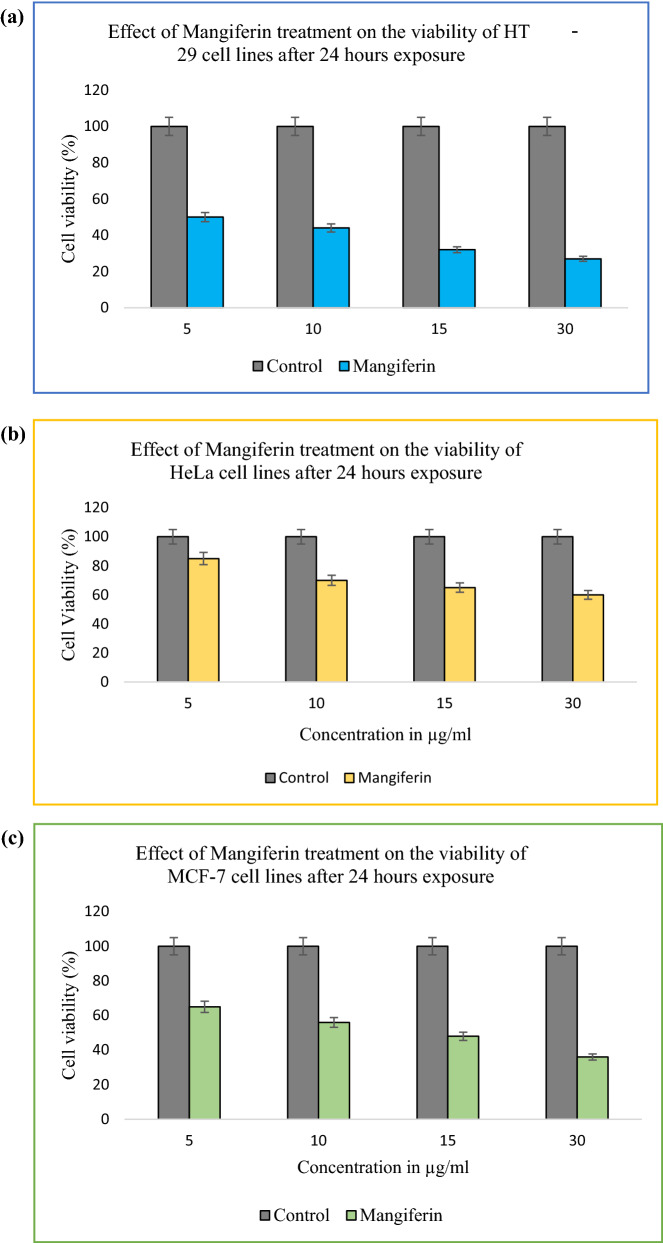
**a** Dose-dependent effect of Mangiferin on HT 29 cell lines; **b** Dose-dependent effect of Mangiferin on HeLa cell lines; **c** Dose-dependent effect of Mangiferin on MCF 7 cell lines. Plots are mean with standard deviation. (n = 3). The data values are statistically significant

Through in silico analysis showed that Mangiferin has significant binding efficiency with COX-2 protein, hence mangiferin efficacy mainly in the treatment of colorectal cancer is evaluated in the present study. Mangiferin possesses antitumor activity and can affect immune function [5–8]. The anticancer potential of Mangiferin treatment on various cancer cell lines such as colorectal cancer (HT 29), cervical cancer (HeLa), and breast cancer (MCF 7) cell lines were evaluated in the present study. The cell viability after 24, hr treatments is presented in Fig. 5.

Mangiferin inhibited the cell proliferation of all the cancer cell lines in a dose-dependent manner. However, the inhibition was more prominent in colorectal cancer cell lines (Fig. 5). Several studies reported that the COX-2 expression was elevated in colorectal tumors, in comparison to normal colorectal tissue. Numerous studies found an over-expression of COX-2 leads to colorectal tumors [42–47].

When HT 29 cells were treated with Mangiferin, the proliferation of HT 29 cells was significantly (P < 0.05) reduced compared with that of the control group (non-treated group) (Fig. 5). After 24 h treatment, the proliferation rate was reduced to 32% for 75 µg/ml of MBN treatment and 27% for 30 µg/ml of Mangiferin treatment (Fig. 5). In the current study, in silico analysis already showed the selective binding of mangiferin with Cox-2 protein, hence this could be concluded mangiferin would be effective in the treatment of colorectal cancer.

However, when HeLa and MCF 7 cells were treated with mangiferin, similarities in results were observed, although, the inhibition rate was more pronounced in the case of HT 29 cells. The proliferation of HeLa cells and MCF 7 cells was significantly (P < 0.005) reduced compared with that of the control group (non-treated group) (Fig. 5). After 24 h treatment, the proliferation rate was reduced to 60%, and 36% in HeLa and MCF 7 cell lines respectively.

The dose-dependent inhibition effects on cell growth from Mangiferin were observed on 24 h treatment of cancer cell lines. However, Mangiferin has shown significantly lower cytotoxicity towards HeLa cell lines (Fig. 5). Few studies have shown that bioactive compounds have a preferential selection of killing cancer cells [11–15, 48–52]. The present study has shown that the cytotoxic effects of Mangiferin were more effective in colorectal cancer and breast cancer cell lines than cervical cancer cell lines (Fig. 5). In comparison with the data of the present study, Mangiferin has a higher inhibition rate to colorectal cancer cell line (Fig. 5), therefore it is suggested that mangiferin blocks the overexpression of COX-2 in colorectal carcinomas. This could plead for the potential use of Mangiferin as a COX-2 inhibitor drug. MGF-induced COX-2 protein inhibition has been suggested to be an important mechanism for cancer prevention.

## Conclusion

Molecular docking of mangiferin with various colon cancer target proteins was carried out to elucidate its mode of action. The efficiency and frequency of amino acid interaction with mangiferin were also analyzed. Mangiferin expressed maximum interaction with lysine, arginine, and asparagine and the least interaction with methionine and isoleucine with the target proteins. Further, it can be concluded that mangiferin interacted more with amino acids containing nonpolar R-group, polar uncharged R-group, and positively charged R-group and least with amino acids containing negatively charged R groups. Docking analysis showed that mangiferin had a higher affinity towards enzymes involved in Arachidonic acid (AA) metabolism as the binding score was maximum for LTA4H protein (− 10.3 kcal/mol) and COX-2(− 10.1 kcal/mol). Proteins associated with Akt, TNFα, NF-қB, and Wnt/β catenin pathways were docked with mangiferin and the result showed that mangiferin can block the hyperactivation of, these pathways. Docking analysis showed that mangiferin can target multiple enzymes/proteins involved in the progression of CRC which makes it a potential candidate in the development of the novel drug for the treatment of CRC. Pharmacophore Analysis of mangiferin using PharmaGist software generated a model that was identical with the model generated for five COX-2 inhibitor drugs, indicating the possibility of mangiferin blocking the activity of the COX-2 enzyme. Toxicity analysis of mangiferin was evaluated using T.E.S.T software and was predicted to be non-toxic and not bioaccumulate. Mangiferin has a higher cytotoxic effect on colorectal cancer cell lines, therefore it is suggested that mangiferin blocks the overexpression of COX-2 in colorectal carcinomas. This could plead for the potential use of Mangiferin as a COX-2 inhibitor drug. It can be concluded that Mangiferin may be a promising candidate for the site-directed target in the future for the treatment of colorectal cancer.

## Supplementary Information

**Additional file 1: Figure S1.** Binding of Rofecoxib inhibitor with chain A of COX-2. Note: binding was present in the crystal structure of COX-2. **Figure S2.** Binding of Rofecoxib inhibitor with chain B of COX-2. Note: binding was present in the crystal structure of COX-2. **Figure S3.** (a-m). Pose comparisons of along with bounded RCX ligand for pose comparison. **Figure S4.** Symbols used to show Protein–Ligand Interaction (LigPlus Software). **Figure S5.** Protein–Ligand Interaction of RCX ligand with COX-2 chain A. **Figure S6.** Protein–Ligand Interaction of Pose 01 of molecular docking of Mangiferin at the site of RCX inhibitor ligand. **Figure S7.** Protein–Ligand Interaction of Pose 02 of molecular docking of Mangiferin at the site of RCX inhibitor ligand. **Figure S8.** Protein–Ligand Interaction of Pose 03 of molecular docking of Mangiferin at the site of RCX inhibitor ligand. **Figure S9.** Protein–Ligand Interaction of Pose 04 of molecular docking of Mangiferin at the site of RCX inhibitor ligand. **Figure S10.** Protein–Ligand Interaction of Pose 05 of molecular docking of Mangiferin at the site of RCX inhibitor ligand. **Figure S11.** Protein–Ligand Interaction of RCX ligand with COX-2 chain B. **Figure S12.** Protein–Ligand Interaction of Pose 01 of molecular docking of Mangiferin at the site of RCX inhibitor ligand. **Figure S13.** Protein–Ligand Interaction of Pose 02 of molecular docking of Mangiferin at the site of RCX inhibitor ligand.

**Additional file 2: Table S1.** Grid Map of Target Proteins. **Table S2.** Area and volume predicted by CASTp for each protein (as active sites). **Table S3.** 2D-QSAR models for Xanthone derivatives. **Table S4.** Pearson correlation matrix of selected descriptors along with pIC50 value. **Table S5.** Toxicity Classes.

## Data Availability

All data generated or analyzed during the study are included in this manuscript and supplementary files. Any further data required are available from the corresponding author on reasonable request.
