# Supplementary material for: In-silico and in-vitro studies on the efficacy of mangiferin against colorectal cancer
Source: BMC Chem. 2022 Jun 7;16(1):42. doi: 10.1186/s13065-022-00835-9 (PMC9172119; doi:10.1186/s13065-022-00835-9)
Supplement: Supplementary file 1 — Additional file 1: Figure S1. Binding of Rofecoxib inhibitor with chain A of COX-2. Note: binding was present in the crystal structure of COX-2. Figure S2. Binding of Rofecoxib inhibitor with chain B of COX-2. Note: binding was present in the crystal structure of COX-2. Figure S3. (a-m). Pose comparisons of along with bounded RCX ligand for pose comparison. Figure S4. Symbols used to show Protein–Ligand Interaction (LigPlus Software). Figure S5. Protein–Ligand Interaction of RCX ligand with COX-2 chain A. Figure S6. Protein–Ligand Interaction of Pose 01 of molecular docking of Mangiferin at the site of RCX inhibitor ligand. Figure S7. Protein–Ligand Interaction of Pose 02 of molecular docking of Mangiferin at the site of RCX inhibitor ligand. Figure S8. Protein–Ligand Interaction of Pose 03 of molecular docking of Mangiferin at the site of RCX inhibitor ligand. Figure S9. Protein–Ligand Interaction of Pose 04 of molecular docking of Mangiferin at the site of RCX inhibitor ligand. Figure S10. Protein–Ligand Interaction of Pose 05 of molecular docking of Mangiferin at the site of RCX inhibitor ligand. Figure S11. Protein–Ligand Interaction of RCX ligand with COX-2 chain B. Figure S12. Protein–Ligand Interaction of Pose 01 of molecular docking of Mangiferin at the site of RCX inhibitor ligand. Figure S13. Protein–Ligand Interaction of Pose 02 of molecular docking of Mangiferin at the site of RCX inhibitor ligand. [file 13065_2022_835_MOESM1_ESM.pdf]

Sl No.	Protein Receptors	PDB ID	Grid Size			Grid Center		
			**X**	**Y**	**Z**	**X**	**Y**	**Z**
1	APC	3NMZ	74	80	76	39.464	-35.473	-15.899
2	BUBR1	3SI5	54	34	50	18.288	-1.496	30.564
3	CDK8	3RGF	78	60	74	0.004	4.816	14.09
4	CK2α	3WAR	64	62	58	-6.857	-10.782	17.708
5	FABP6	5L8I	48	40	44	50.711	-7.935	59.08
6	K-Ras	4OBE	46	40	40	-5.975	-22.372	42.093
7	SPINDLE ASSEMBLY CHECKPOINT PROTEIN HUMAN MAD2	1DUJ	62	48	48	-14.163	27.893	-2.86
9	Bcl-xL	1MAZ	48	42	46	1.936	22.227	39.275
10	Bcl-2	2XA0	40	44	36	33.475	-16.507	-15
12	COX-2	3NT1	50	52	40	-33.808	-45.335	-24.417
14	CYTOCHROME P450	4NZ2	70	70	54	-39.032	-61.36	-25.174
15	PRTOEIN KINASE B	1UNR	64	62	88	12.956	-0.73	-1.799
16	TNFα	4TWT	62	74	66	5.591	95.39	234.344
17	LEUKOTRIENE A4 HYDROLASE	1HS6	76	74	62	16.502	40.003	64.68
18	NF-κB	1VKX	74	84	90	0.144	37.93	57.762

Table **S1**. Grid Map of Target Proteins.

Sl No.	Protein Receptors	Area	Volume
1	APC	30554.495	19137.348
2	BUBR1	929.352	4503.129
3	CDK8	505.676	453.661
4	CK2α	22373.545	13180.014
5	FABP6	6460.869	4383.774
6	K-Ras	9880.206	5579.96
7	SPINDLE ASSEMBLY CHECKPOINT PROTEIN HUMAN MAD2	650.976	407.29
9	Bcl-xL	8154.042	3980.534
10	Bcl-2	9510.191	5132.427
12	COX-2	2009.530	1567.961
14	CYTOCHROME P450	32433.279	21039.17
15	PRTOEIN KINASE B	5370.878	2791.758
16	TNFα	15321.63	9131.527
17	LEUKOTRIENE A4 HYDROLASE	1489.538	1170.915
18	NF-κB	31569.852	21777.641

Table **S2.** Area and volume predicted by CASTp for each protein(as active sites).

Model No	Regression Equation
1	pIC_{50} = - 3.7345 (± 2.5333) MATS7m - 1.2090 (± 1.6292) IC5 + 10.3389 (± 8.1164) (n = 11; R = 0.890; s = 0.187; F = 15.322; R^2= 0.7930, R^2-Adj.= 0.7412; p = 0.0018; Q^2 = 0.563; S_{Press} = 0.272 ; S_{DEP} = 0.243)
**2**	**pIC_{50} = + 7.4923 (± 5.3523) MATS1e - 2.5401 (± 2.2482) MATS7e + 5.1776 (± 0.5934) (n = 11; R = 0.907; s = 0.173; F = 18.502; R^2 = 0.8222; R^2-Adj.= 0.7778; p = 0.0010; Q^2 = 0.615; S_{Press} = 0.255; S_{DEP} = 0.228)**
3	pIC_{50} = + 7.7926 (± 5.2314) MATS1e - 2.4613 (± 2.1907) MATS7v + 5.2097 (± 0.5738) (n = 11; R = 0.906; s = 0.174; F = 18.386; R^2 = 0.8213; R^2-Adj. = 0.7766; p = 0.0010; Q^2 = 0.587 ; SPress = 0.264 ; S_{DEP} = 0.236)
4	pIC_{50} = + 8.0187 (± 6.4297) MATS1e - 0.7222 (± 0.9798) H6u + 6.4023 (± 1.0392) (n = 11; R = 0.871; s = 0.202; F = 12.569; R^2= 0.7586; R^2-Adj.= 0.6982; p = 0.0034; Q^2 = 0.644; S_{Press} = 0.245; S_{DEP} = 0.220)
5	pIC_{50} = + 1.7703 (± 2.2997) GATS7v + 0.0957 (± 0.1267) DISPv + 2.2872 (± 2.1511) (n = 11; R = 0.811; s = 0.241; F = 7.672; R^2= 0.6573; R^2-Adj.= 0.5716; p = 0.0138; Q^2 = 0.374; S_{Press} = 0.325 ; S_{DEP} = 0.291)
6	pIC_{50} = - 2.9042 (± 2.9745) MATS7e + 0.0892 (± 0.1150) DISPv + 3.9688 (± 0.6540) (n = 11;R = 0.841; s = 0.222; F = 9.680; R^2= 0.7076; R^2-Adj.= 0.6345; p = 0.0073; Q^2 = 0.495; S_{Press} = 0.292; S_{DEP} = 0.261)
7	pIC_{50} = - 2.9872 (± 3.1624) MATS7e - 1.3748 (± 2.0869) IC5 + 11.2582 (± 10.3893) (n = 11; R = 0.826; s = 0.232; F = 8.592; R^2= 0.6823;R^2-Adj.= 0.6029;p = 0.0102; Q^2 = 0.496; S_{Press} = 0.292; S_{DEP} = 0.261)
8	pIC_{50} = - 2.7542 (± 2.9422) MATS7v + 0.0954 (± 0.1141) DISPv + 3.9442 (± 0.6607) (n = 11; R = 0.836; s = 0.226; F = 9.251; R^2= 0.6981; R^2-Adj.= 0.6227; p = 0.0083; Q^2 = 0.433; S_{Press} = 0.310; S_{DEP} = 0.277)

Table **S3.** 2D-QSAR models for Xanthone derivatives.

	**pIC_{50}**	**MATS7m**	**MATS1e**	**GATS7m**	**GATS7v**	**GATS7e**	**GATS1e**	**MATS7e**	**MATS7v**	**DISPp**	**DISPv**	**H6u**	**HATS7p**	**IC5**
**pIC_{50}**	1.000	0.847	0.819	0.847	0.726	0.744	0.831	0.769	0.747	0.749	0.723	0.713	0.707	0.703
**MATS7m**	0.847	1.000	0.733	0.926	0.779	0.830	0.805	0.927	0.881	0.679	0.701	0.752	0.754	0.561
**MATS1e**	0.819	0.733	1.000	0.878	0.610	0.632	0.889	0.539	0.503	0.638	0.740	0.576	0.612	0.620
**GATS7m**	0.847	0.926	0.878	1.000	0.862	0.893	0.937	0.847	0.822	0.758	0.785	0.820	0.803	0.653
**GATS7v**	0.726	0.779	0.610	0.862	1.000	0.995	0.864	0.899	0.930	0.724	0.597	0.964	0.885	0.715
**GATS7e**	0.744	0.830	0.632	0.893	0.995	1.000	0.872	0.928	0.947	0.733	0.626	0.958	0.882	0.692
**GATS1e**	0.831	0.805	0.889	0.937	0.864	0.872	1.000	0.762	0.759	0.682	0.707	0.839	0.865	0.739
**MATS7e**	0.769	0.927	0.539	0.847	0.899	0.928	0.762	1.000	0.992	0.665	0.577	0.876	0.827	0.599
**MATS7v**	0.747	0.881	0.503	0.822	0.930	0.947	0.759	0.992	1.000	0.672	0.548	0.905	0.852	0.637
**DISPp**	0.749	0.679	0.638	0.758	0.724	0.733	0.682	0.665	0.672	1.000	0.921	0.757	0.742	0.840
**DISPv**	0.723	0.701	0.740	0.785	0.597	0.626	0.707	0.577	0.548	0.921	1.000	0.645	0.692	0.735
**H6u**	0.713	0.752	0.576	0.820	0.964	0.958	0.839	0.876	0.905	0.757	0.645	1.000	0.921	0.784
**HATS7p**	0.707	0.754	0.612	0.803	0.885	0.882	0.865	0.827	0.852	0.742	0.692	0.921	1.000	0.868
**IC5**	0.703	0.561	0.620	0.653	0.715	0.692	0.739	0.599	0.637	0.840	0.735	0.784	0.868	1.000

Table **S4.** Pearson correlation matrix of selected descriptors along with pIC_{50} value.

**Toxicity Rating**	**Commonly Used Term**	**(single dose to rats) mg/kg**
1	Extremely Toxic	1 or less
2	Highly Toxic	1-50
3	Moderately Toxic	50-500
4	Slightly Toxic	500-5000
5	Practically Non-toxic	5000-15,000
6	Relatively Harmless	15,000 or more

Table **S5.** Toxicity Classes.
