# Supplementary material for: In-silico and in-vitro studies on the efficacy of mangiferin against colorectal cancer
Source: BMC Chem. 2022 Jun 7;16(1):42. doi: 10.1186/s13065-022-00835-9 (PMC9172119; doi:10.1186/s13065-022-00835-9)
Supplement: Supplementary file 2 — Additional file 2: Table S1. Grid Map of Target Proteins. Table S2. Area and volume predicted by CASTp for each protein (as active sites). Table S3. 2D-QSAR models for Xanthone derivatives. Table S4. Pearson correlation matrix of selected descriptors along with pIC50 value. Table S5. Toxicity Classes. [file 13065_2022_835_MOESM2_ESM.pdf]

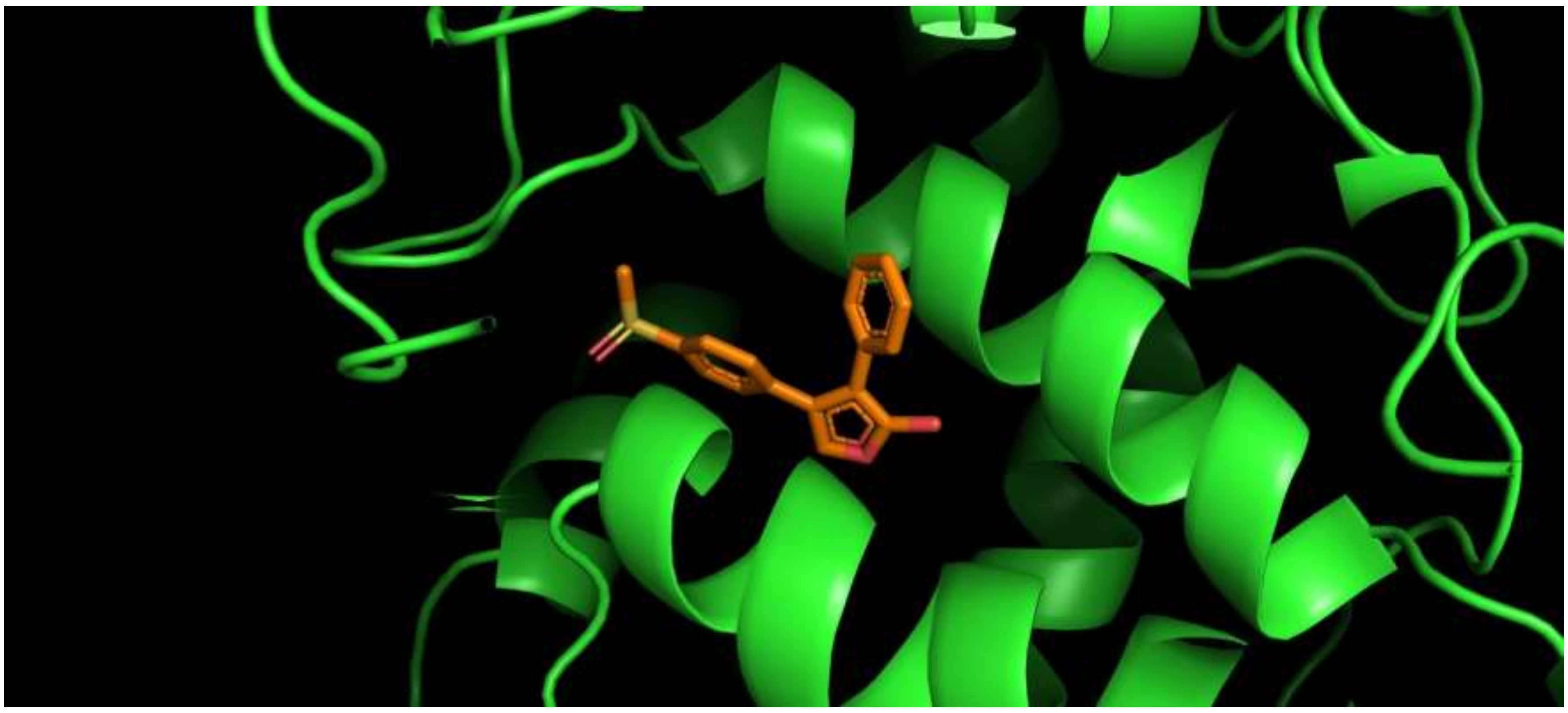

Supplementary Fig 1: Binding of Rofecoxib inhibitor with chain A of COX-2. Note: binding was present in the crystal structure of COX-2 (PDB ID: 5KIR)

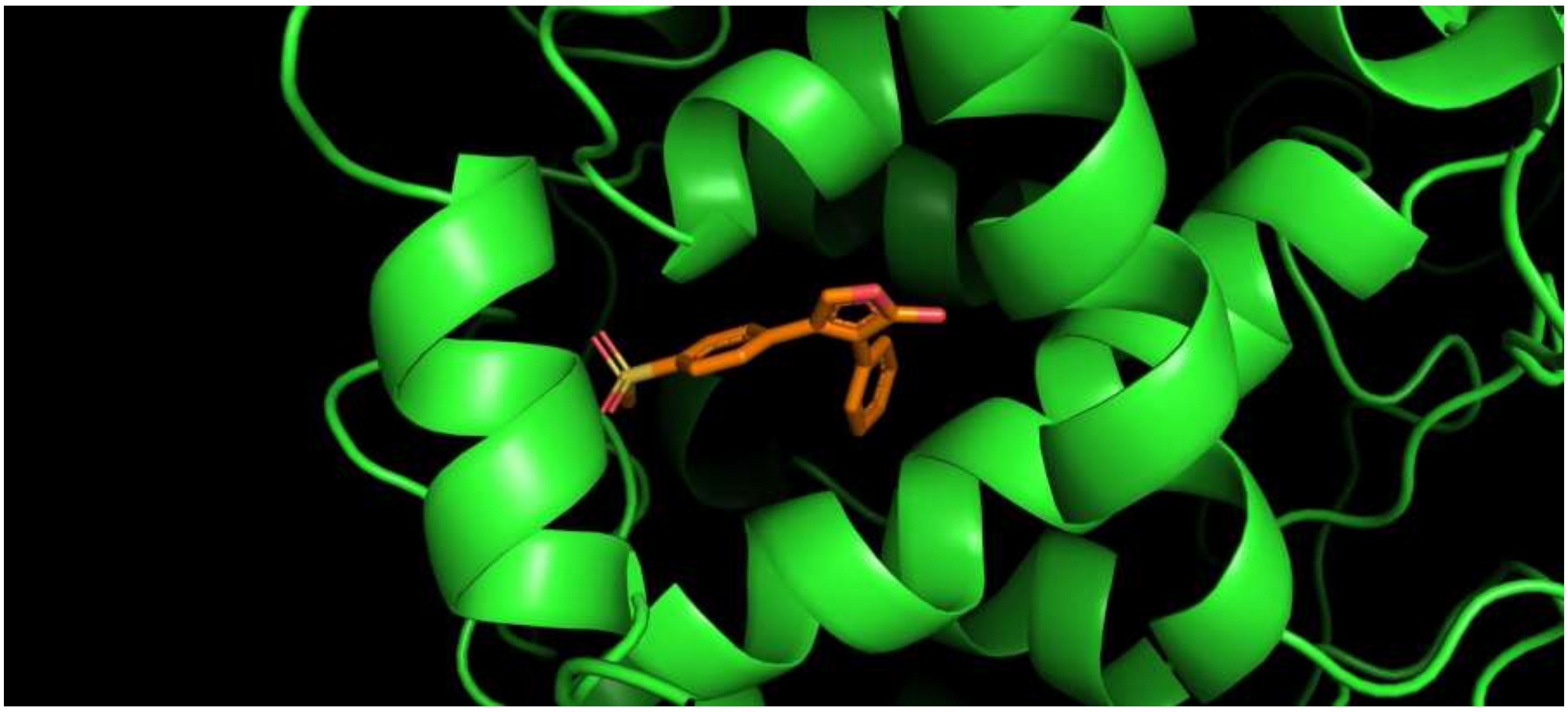

Supplementary Fig 2: Binding of Rofecoxib inhibitor with chain B of COX-2. Note: binding was present in the crystal structure of COX-2 (PDB ID: 5KIR)

a

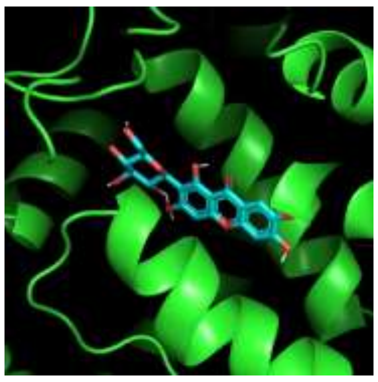

b

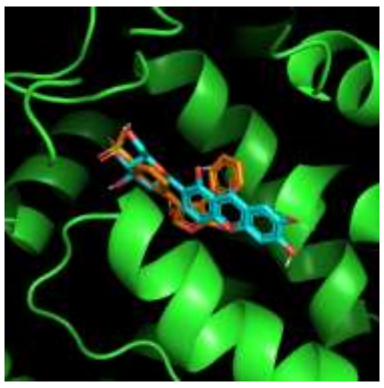

c

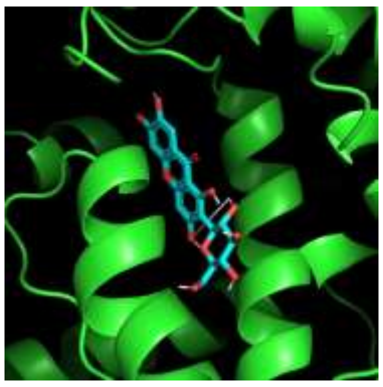

d

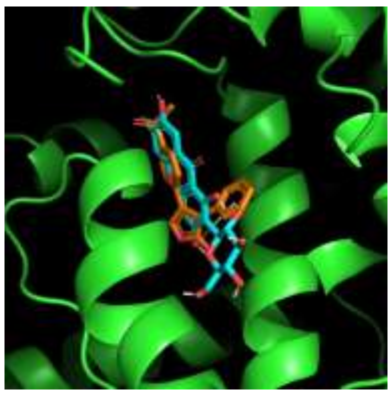

e

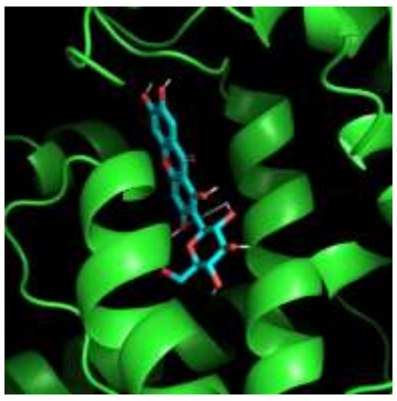

f

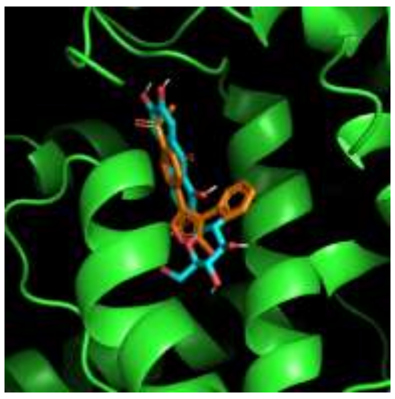

g

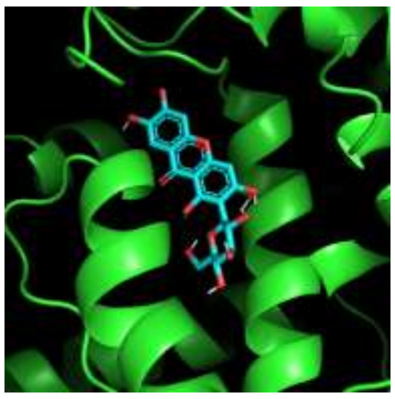

h

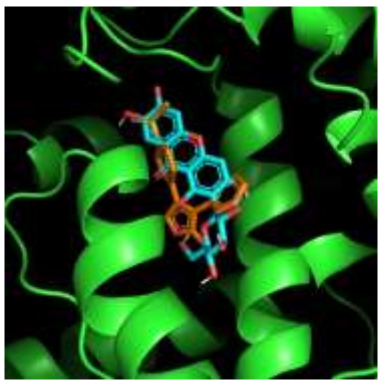

i

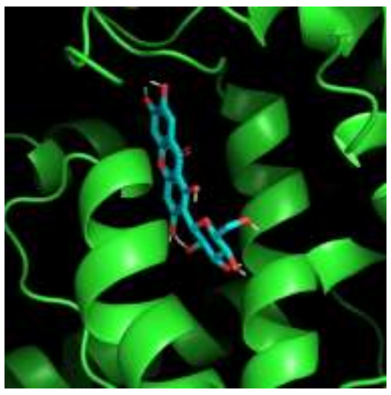

j

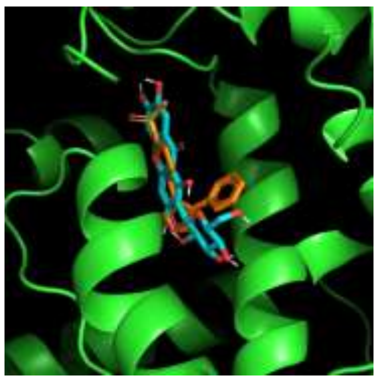

k

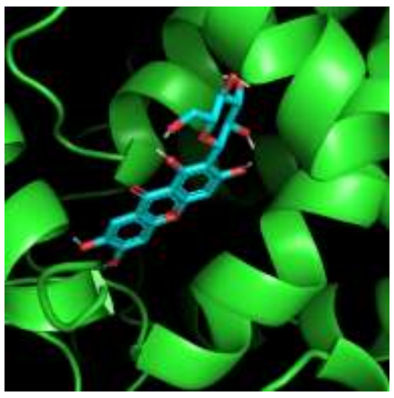

l

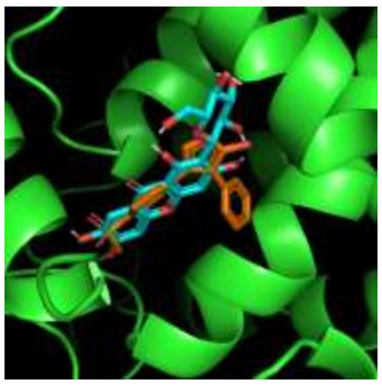

m

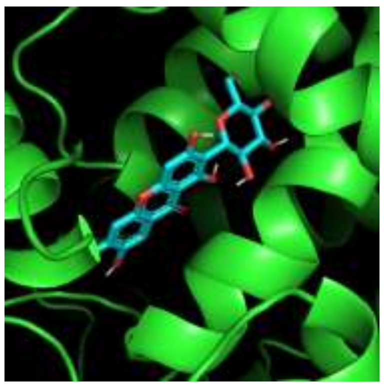

n

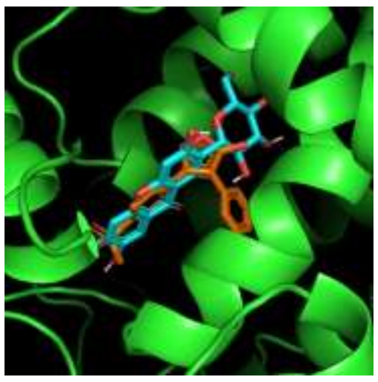

Supplementary Fig 3. (a-m). Pose comparisons of along with bounded RCX ligand for Pose Comparison

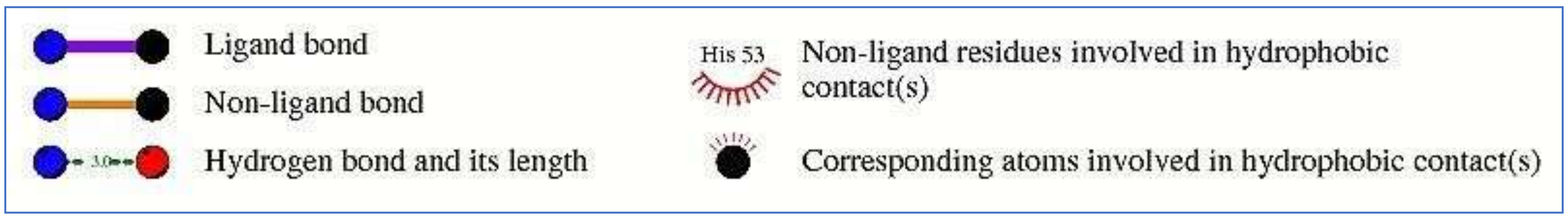

Supplementary Fig 4: Symbols used to show Protein-Ligand Interaction (LigPlus Software).

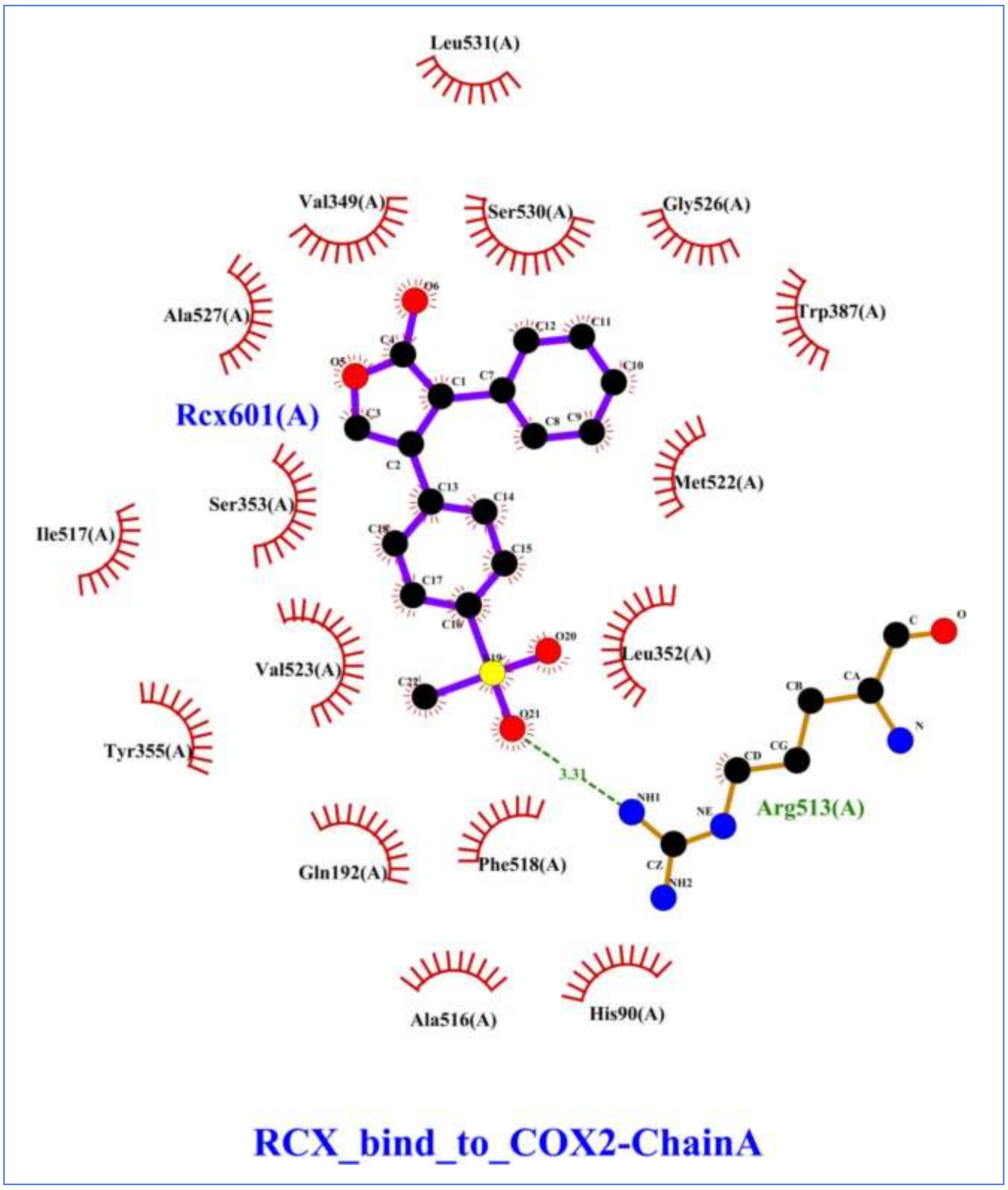

Supplementary Fig 5: Protein-Ligand Interaction of RCX ligand with COX-2 chain A.

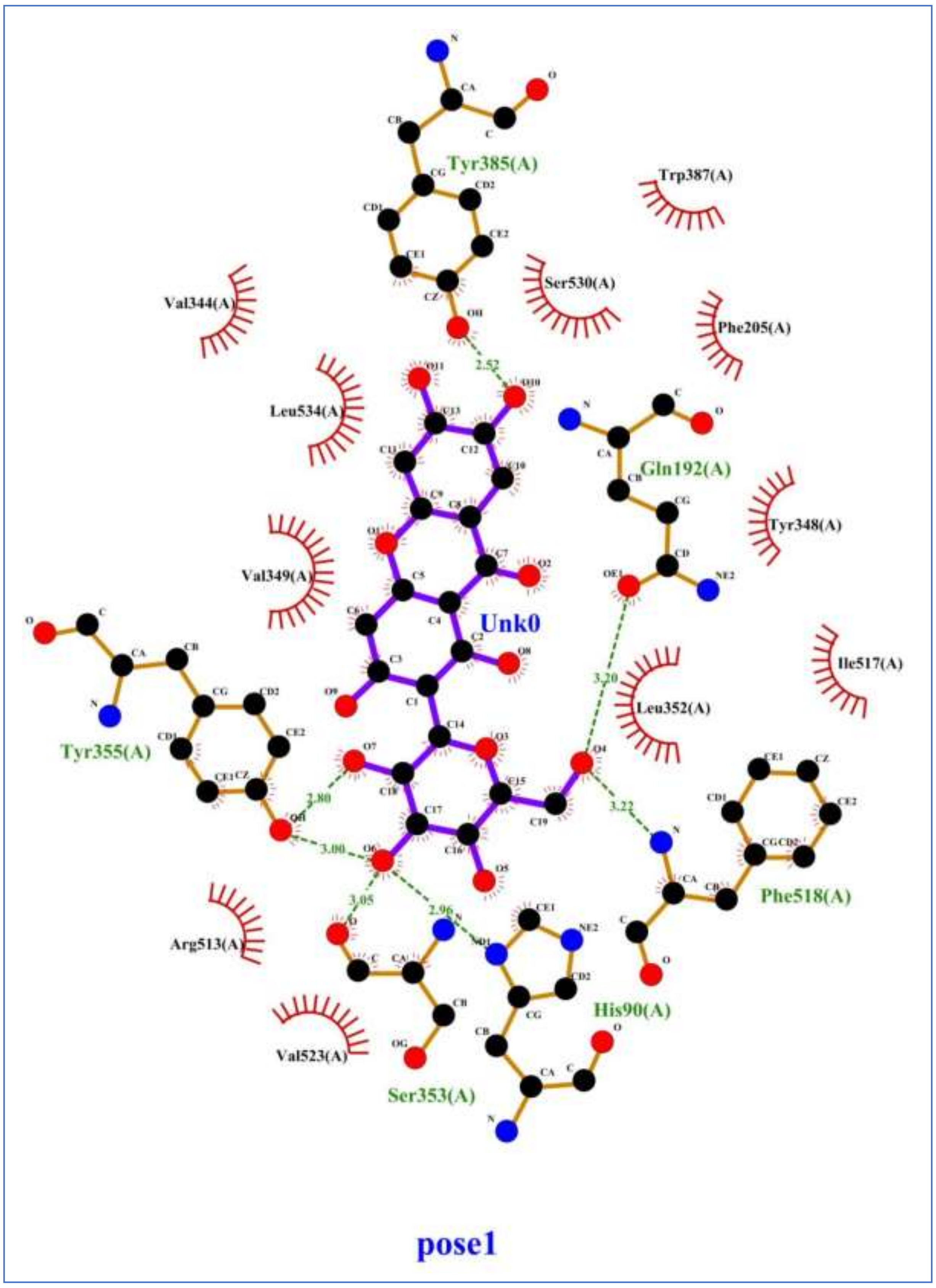

Supplementary Fig 6. Protein-Ligand Interaction of Pose 01 of molecular docking of Mangiferin at the site of RCX inhibitor ligand.

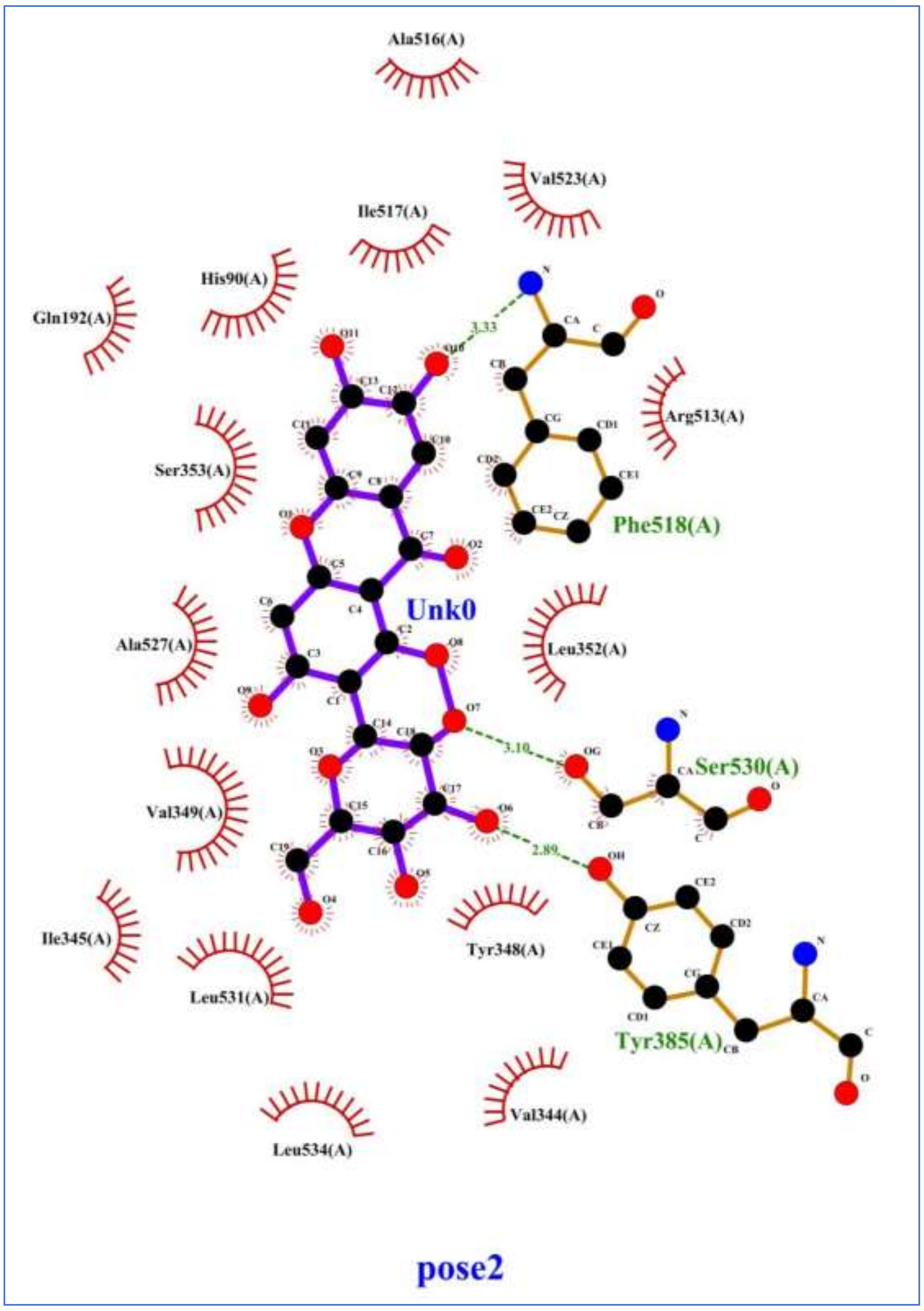

Supplementary Fig 7. Protein-Ligand Interaction of Pose 02 of molecular docking of Mangiferin at the site of RCX inhibitor ligand.

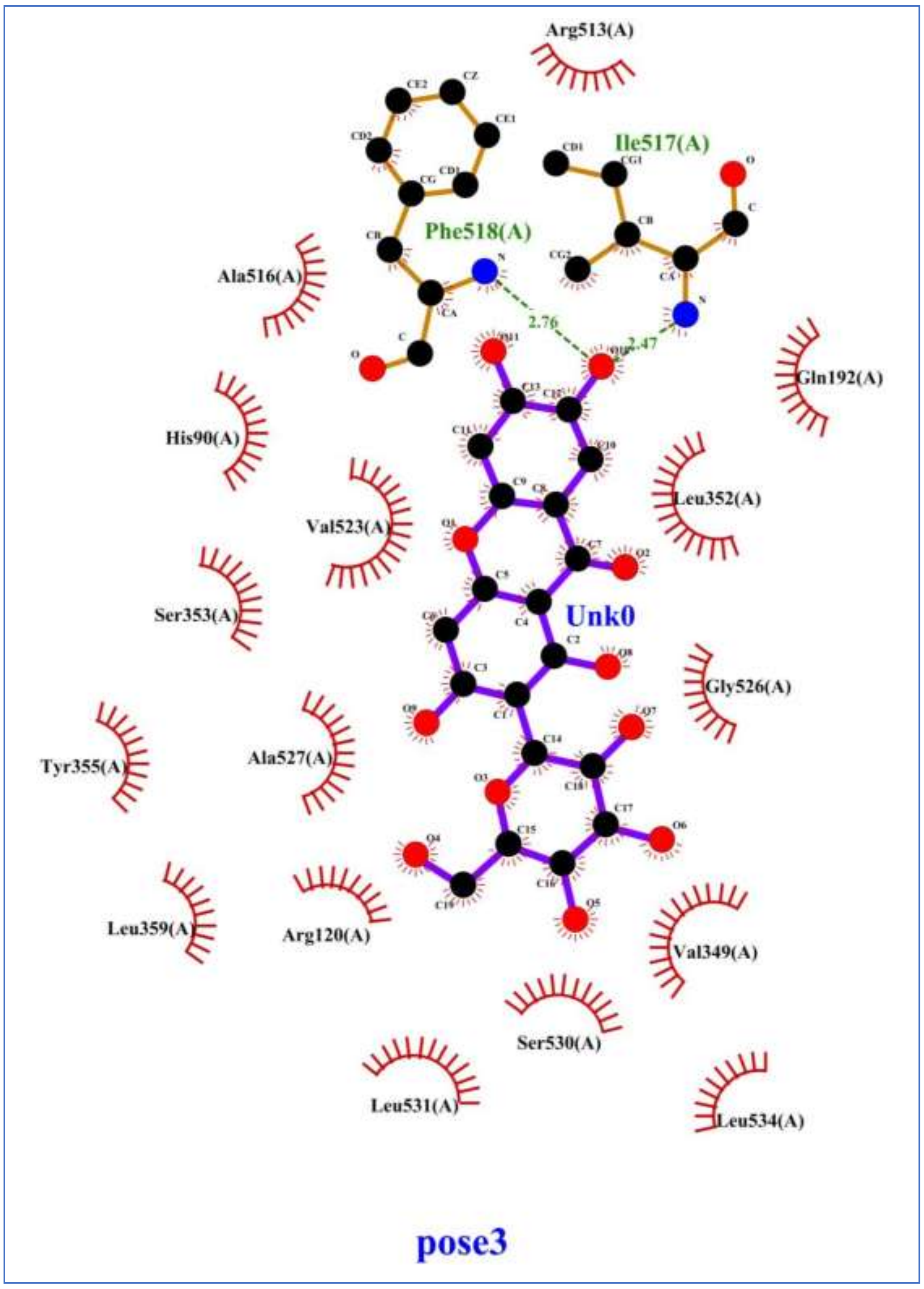

Supplementary Fig 8. Protein-Ligand Interaction of Pose 03 of molecular docking of Mangiferin at the site of RCX inhibitor ligand.

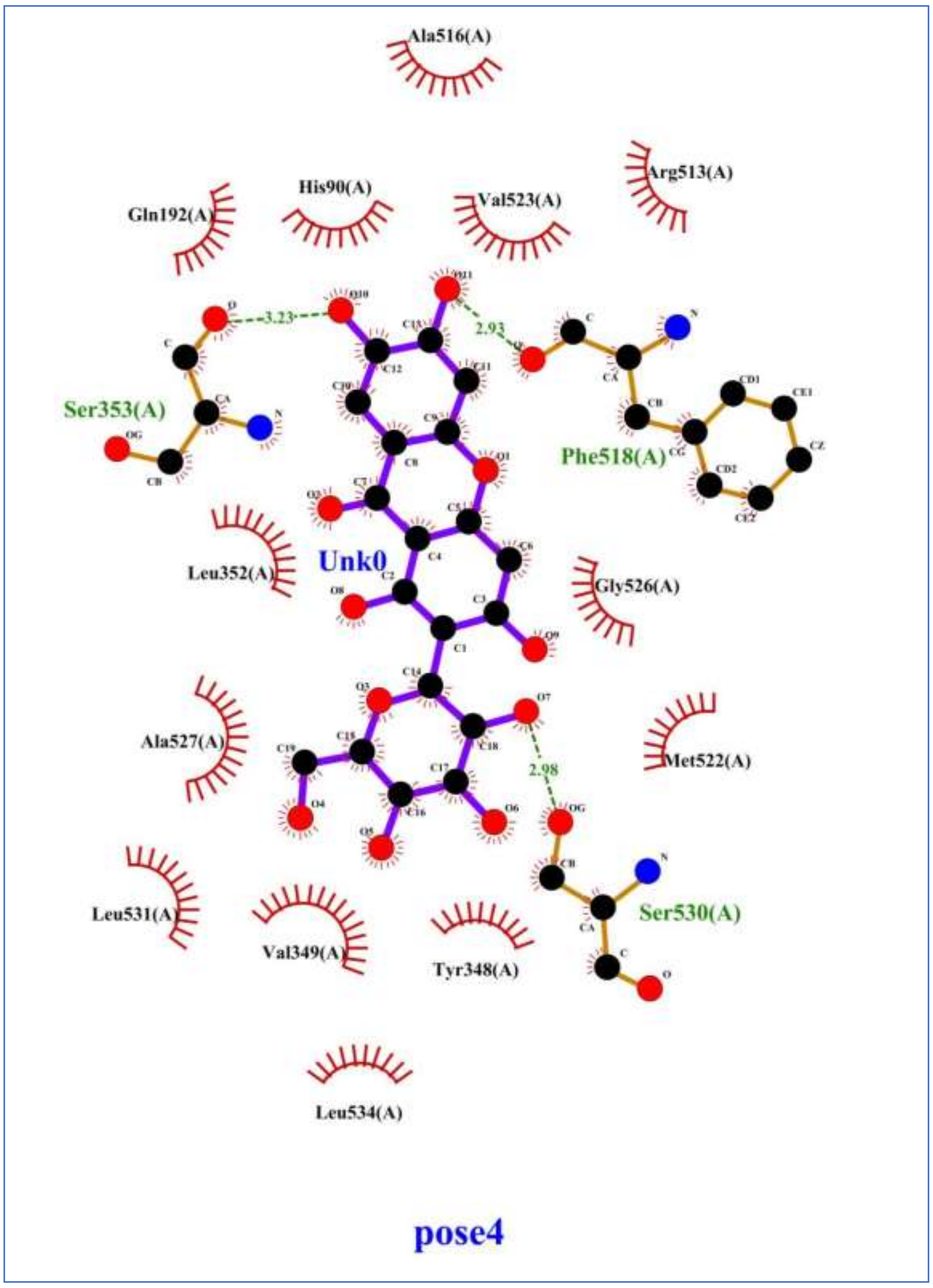

Supplementary Fig 9. Protein-Ligand Interaction of Pose 04 of molecular docking of Mangiferin at the site of RCX inhibitor ligand.

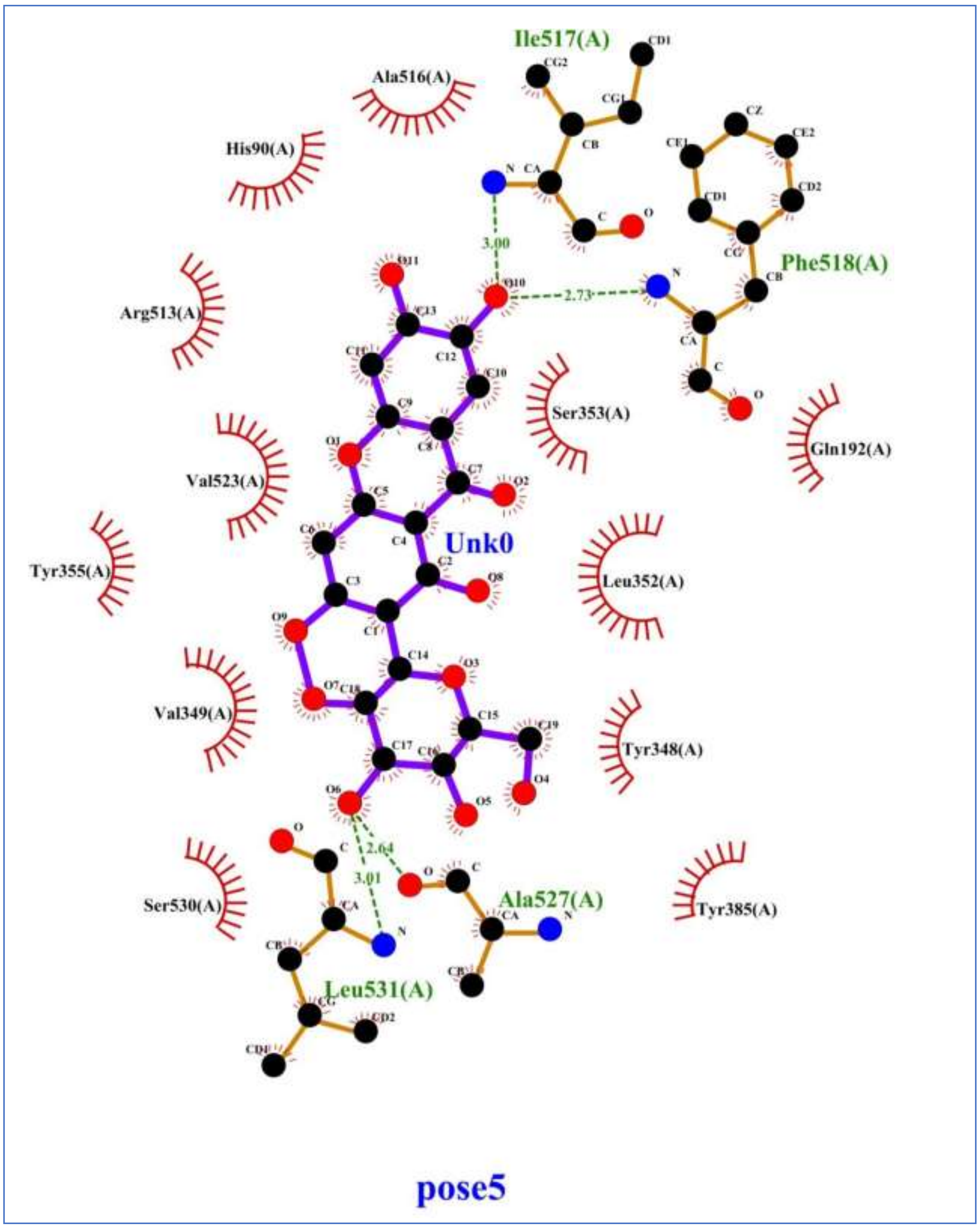

Supplementary Fig 10. Protein-Ligand Interaction of Pose 05 of molecular docking of Mangiferin at the site of RCX inhibitor ligand.

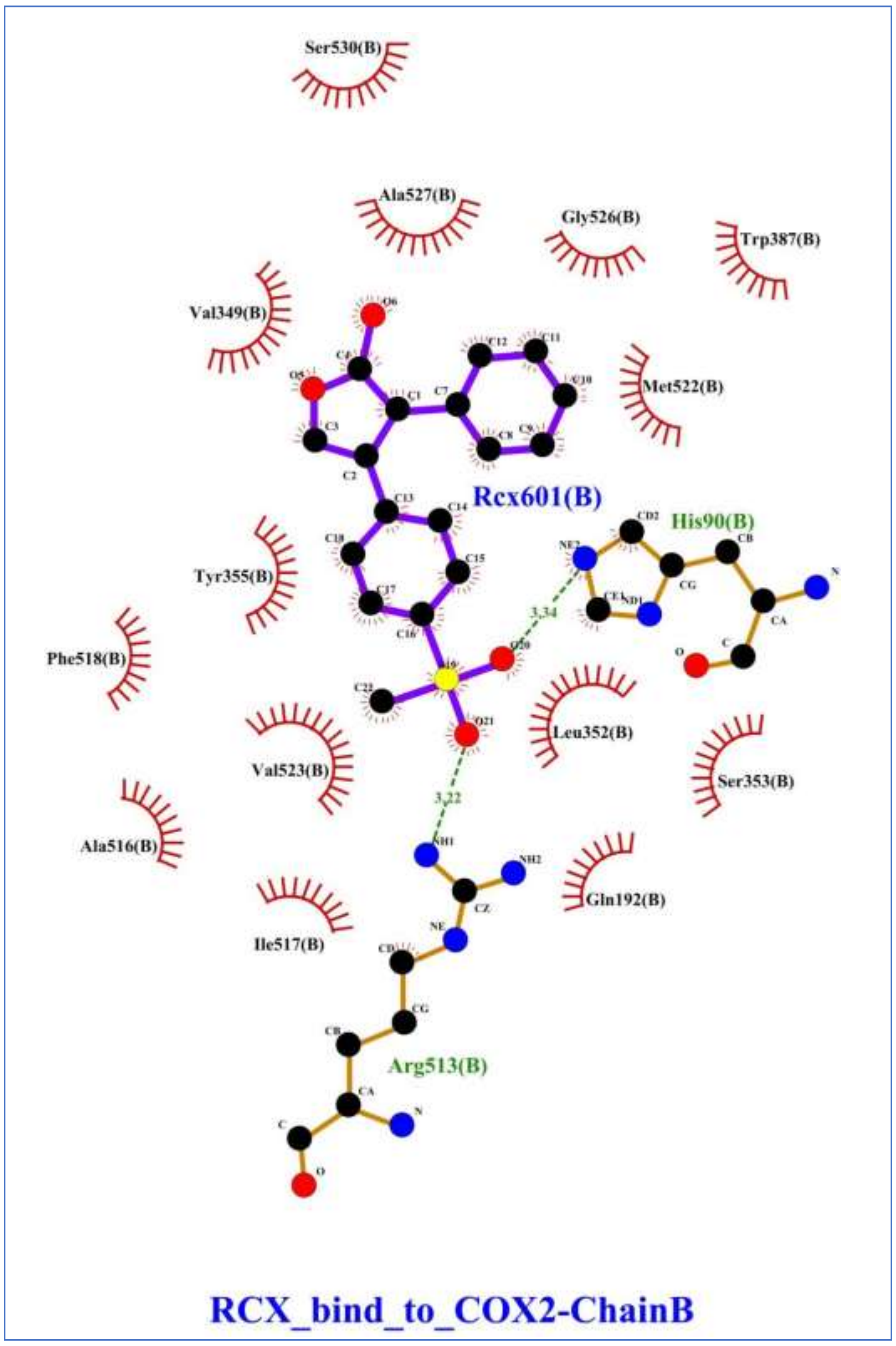

Supplementary Fig 11. Protein-Ligand Interaction of RCX ligand with COX-2 chain B.

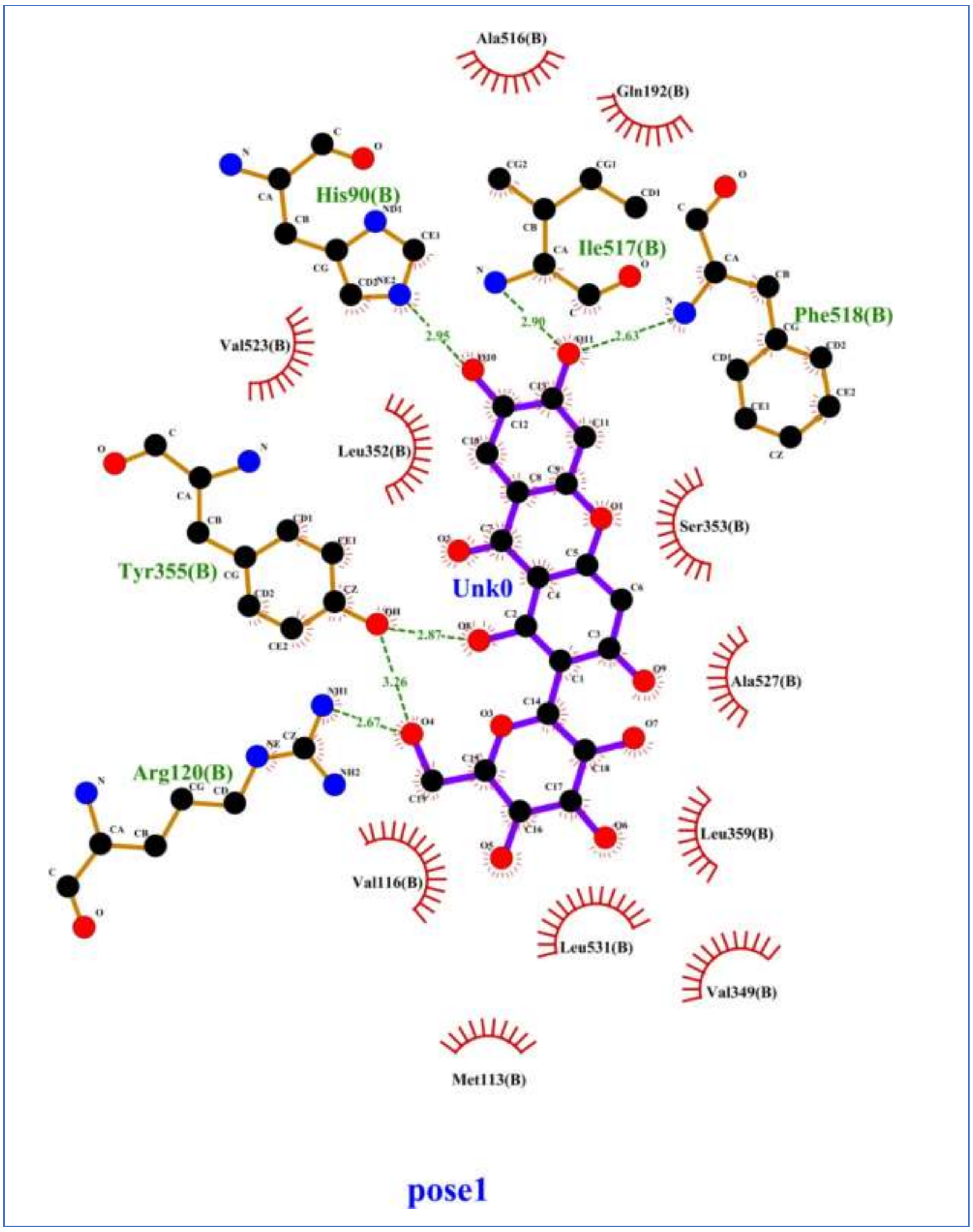

Supplementary Fig 12. Protein-Ligand Interaction of Pose 01 of molecular docking of Mangiferin at the site of RCX inhibitor ligand.

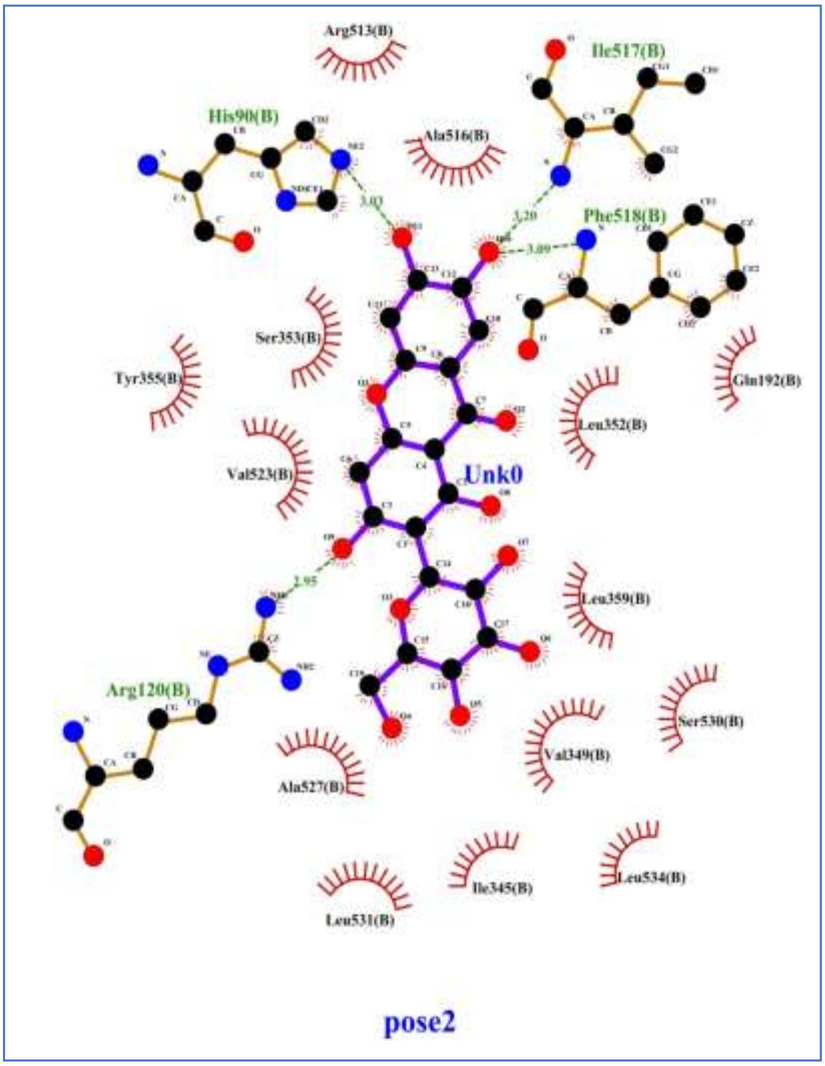

Supplementary Fig 13. Protein-Ligand Interaction of Pose 02 of molecular docking of Mangiferin at the site of RCX inhibitor ligand.
